# The maximum *T*_c_ of conventional superconductors at ambient pressure

**DOI:** 10.1038/s41467-025-63702-w

**Published:** 2025-09-10

**Authors:** Kun Gao, Tiago F. T. Cerqueira, Antonio Sanna, Yue-Wen Fang, Đorđe Dangić, Ion Errea, Hai-Chen Wang, Silvana Botti, Miguel A. L. Marques

**Affiliations:** 1 Research Center Future Energy Materials and Systems of the Research Alliance Ruhr, Bochum, Germany; 2 Faculty of Physics and Astronomy and Interdisciplinary Centre for Advanced Materials Simulation, Ruhr University Bochum, Bochum, Germany; 3 CFisUC, Department of Physics, University of Coimbra, Coimbra, Portugal; 4 Max-Planck-Institut für Mikrostrukturphysik, Halle, Germany; 5 Institut für Physik, Martin-Luther-Universität Halle-Wittenberg, Halle, Germany; 6 Centro de Física de Materiales (CFM-MPC), CSIC-UPV/EHU, Donostia/San Sebastián, Spain; 7 Fisika Aplikatua Saila, University of the Basque Country (UPV/EHU), Donostia/San Sebastián, Spain; 8 Donostia International Physics Center (DIPC), Donostia/San Sebastián, Spain; 9 Interdisciplinary Centre for Advanced Materials Simulation, Ruhr University Bochum, Bochum, Germany

**Keywords:** Superconducting properties and materials, Superconducting properties and materials

## Abstract

The theoretical maximum critical temperature (*T*_c_) for conventional superconductors at ambient pressure remains a fundamental question in condensed matter physics. Through analysis of electron-phonon calculations for over 20,000 metals, we critically examine this question. We find that while hydride metals can exhibit maximum phonon frequencies of more than 5000 K, the crucial logarithmic average frequency ${\omega }_{\log }$ rarely exceeds 1800 K. Our data reveals an inherent trade-off between ${\omega }_{\log }$ and the electron-phonon coupling constant *λ*, suggesting that the optimal Eliashberg function that maximizes *T*_c_ is unphysical. Based on our calculations, we identify Li_2_AgH_6_ and its sibling Li_2_AuH_6_ as theoretical materials that likely approach the practical limit for conventional superconductivity at ambient pressure. Analysis of thermodynamic stability indicates that compounds with higher predicted *T*_c_ values are increasingly unstable, making their synthesis challenging. While fundamental physical laws do not strictly limit *T*_c_ to low-temperatures, our analysis suggests that achieving room-temperature conventional superconductivity at ambient pressure is extremely unlikely.

## Abstract (web summary)

The authors reveal an inherent trade-off between logarithmic average phonon frequency and the electron-phonon coupling constant in conventional BCS superconductors. The analysis suggests that achieving room-temperature conventional superconductivity at ambient pressure is extremely unlikely.

## Introduction

Since the groundbreaking discovery of superconductivity in mercury at 4.2 K in 1911^1^, superconductors have revolutionized both fundamental physics and technological applications. These materials have enabled transformative technologies including magnetic resonance imaging machines, magnetic levitation trains, and ultra-sensitive quantum devices such as SQUIDs. The quest for high-temperature superconductivity remains one of the grand challenges of solid-state physics, as raising the critical temperature would unlock unprecedented possibilities in power transmission, quantum computing, and particle acceleration.

Superconducting materials are traditionally divided into conventional and unconventional. In the former, the topic of the current work, the mechanism responsible for superconductivity is the electron-phonon interaction, electrons form Cooper pairs in a singlet state, and the energy gap has s-wave symmetry. These compounds are well understood within the Bardeen, Cooper, and Schrieffer (BCS) theory^2^, or its strong coupling generalization, Eliashberg theory^3^.

In the 1960s and 1970s, it was believed that the maximum *T*_c_for conventional superconductors would likely be in the range of 30–40 K. This was based on the observed properties of known superconducting materials and the theoretical limits imposed by the electron-phonon interaction. The argument was that to achieve a higher *T*_c_ would require an unrealistically strong electron-phonon coupling or an exceptionally high density of states, both of which seemed improbable given the materials known at the time. These beliefs were supported by experimental data. For example, elemental superconductors like Pb and Nb have *T*_c_= 7.2 K and *T*_c_= 9.25 K, respectively^4^, while more complex compounds like Nb_3_Sn or Nb_3_Ge reach *T*_c_= 17.9 K and *T*_c_= 21.8 K respectively^5^.

Currently, the compound with record conventional superconductivity at room pressure is MgB_2_, with a transition temperature of *T*_c_ = 39 K^6^. This compound exhibits two nearly noninteracting bands of different dimensionality^7^, leading to the coexistence of two energy gaps in the same material^8^. The idea of multiple gaps coexisting in a single superconductor had been explored previously^9,10^, but MgB_2_ stands out as the first system where this phenomenon is so prominently and distinctly manifested^11^.

An alternative approach to achieving high-temperature superconductivity involves the chemical precompression of the hydrogen sub-lattice in hydrides materials^12^. This led to groundbreaking discoveries of very high-*T*_c_ superconductors, such as H_3_S^13–15^, LaH_10_^16–19^, YH_*x*_^20–22^, or even in ternary compounds such as LaBeH_8_, (La,Y)H_10_, (La,Ce)H_9_, etc.^23–28^. Unfortunately, all these systems require very high pressures, seriously limiting their application to technology. A key question in this field is what is the upper limit to the critical temperature of superconductors^29–35^.

In this work, we critically examine this question. Through analysis of electron-phonon calculations for over 20 000 metals we find an inherent trade-off between logarithmic average phonon frequency (${\omega }_{\log }$) and the electron-phonon coupling constant (*λ*), suggesting that optimal Eliashberg functions for maximizing *T*_c_ are unphysical.

## Results and Discussion

Based on the observation that the coupling constant depends mainly on the phonon frequencies, McMillan in 1968^36^ already derived an approximate expression of ${T}_{{{\rm{c}}}}^{\max }$. His result, however, only holds for a *given class of materials* and therefore does not provide an absolute value for this quantity. Further insight can be obtained by maximizing *T*_c_ given by Allen-Dynes’ version of McMillan’s formula^37^

1 $${T}_{{{\rm{c}}}}^{{{\rm{McMillan}}}}=\frac{{\omega }_{\log }}{1.20}\exp \left(-1.04\frac{1+\lambda }{\lambda -{\mu }^{*}(1+0.62\lambda )}\right),$$

where μ* is the Coulomb pseudopotential, *λ* is the electron-phonon coupling constant

2 $$\lambda=2 \int_{0}^{+\infty }\frac{{\alpha }^{2}F(\omega )}{\omega }d\omega,$$

and *α*^2^*F*(*ω*) is the Eliashberg spectral function, calculated from the electron-phonon coupling matrix elements. With ${\omega }_{\log }$ we indicate the logarithmic average of the phonon frequencies:

3 $${\omega }_{\log }=\exp \left[\frac{2}{\lambda }\int_{0}^{+\infty }\ln (\omega )\frac{{\alpha }^{2}F(\omega )}{\omega }d\omega \right].$$

By setting, for simplicity, the Coulomb pseudopotential μ* to zero, one obtains that the maximum is attained at *λ* ≈ 2. However, McMillan’s formula is only valid for values of *λ* ≲ 1.5, while no maximum value for *T*_c_ exists in the original Eliashberg theory^29^. It is also frequently argued that the value of *λ* is limited, as the lattice becomes eventually unstable for very large values of the coupling constant. However, there are some experimentally known superconductors with very high values of *λ*, well above 2.0, both at ambient (e.g., Pb–Bi compounds^38^) and under pressure^39^.

Recent works estimated the maximum *T*_c_ of conventional superconductivity from fundamental limits^40–42^. They all agree on a value of 300–600 K at ambient pressure, suggesting that superconductors may exist at ambient temperature. In the following, we critically look at the basic assumptions used in these estimates. This is done by analyzing experimentally-known superconductors and our calculations of electron-phonon interaction and superconducting properties for more than 20 000 metals^43,44^. This is by far the largest dataset available to date with calculated superconducting properties, and it contains metals with a large variety of chemical elements and crystal structures. It also includes most of the materials that have been proposed in the literature as conventional high-*T*_*c*_ superconductors. These compounds had to be added manually, as in the overwhelming majority of the cases they were thermodynamically highly unstable and were therefore absent from our database. The *σ*-electron systems that we have considered include a series of materials based on MgB_2_ structure^45–56^, on the diamond structure^57–62^, cage-like structures^63–74^, hydrides^34,75–77^, and many other compounds^35,78–82^. In several of these families, we also performed exhaustive machine-learning accelerated^83^ high-throughput calculations that went well beyond what had been reported in the literature. For example, we studied (with a X and Y running over the periodic table) all possible XY_2_ compounds in the structure of MgB_2_; XBC in the structure of LiBC^55^; MgXB_4_ compounds in the structure of MgAlB_4_^56^; all possible B–C phases in the diamond structure for unit cells with up to 12 atoms, all possible Li_*x*_B_*y*_C_*z*_ structures with up to 12 atoms in the unit cell, all XB_3_N_3_ and XYB_6_C_6_ filled sodalite compounds^64^, etc. Finally, this ab initio data was complemented by the investigation, using the machine-learning models developed in refs. ^43,44^, of the superconducting properties of more than 100 million compounds. While no study can be truly exhaustive given the vastness of chemical space, our datasets are comprehensive and extensive, providing us with a thorough overview of conventional superconductivity across materials space.

To estimate the maximum *T*_c_ of conventional superconductors at ambient pressure, the first step is to determine what is the largest phonon frequency ${\omega }_{\max }$ that one can reasonably expect in a realistic compound. Reference^42^ derived the expression

4 $${\omega }_{\max }=\frac{{E}^{{{\rm{electronic}}}}}{\sqrt{m}},$$

where *E*^electronic^ is a typical electronic energy, and *m* is an atomic mass. By inserting a typical *E*^electronic^ = 1 Ry, and the proton mass *m* = *m*^proton^ into the formula, they obtained ${\omega }_{\max }=3680$ K. Compared to the vibrational frequency of H_2_ that is almost 6000 K^84^, this value does not appear to be an overestimation.

**Fig. 1:**
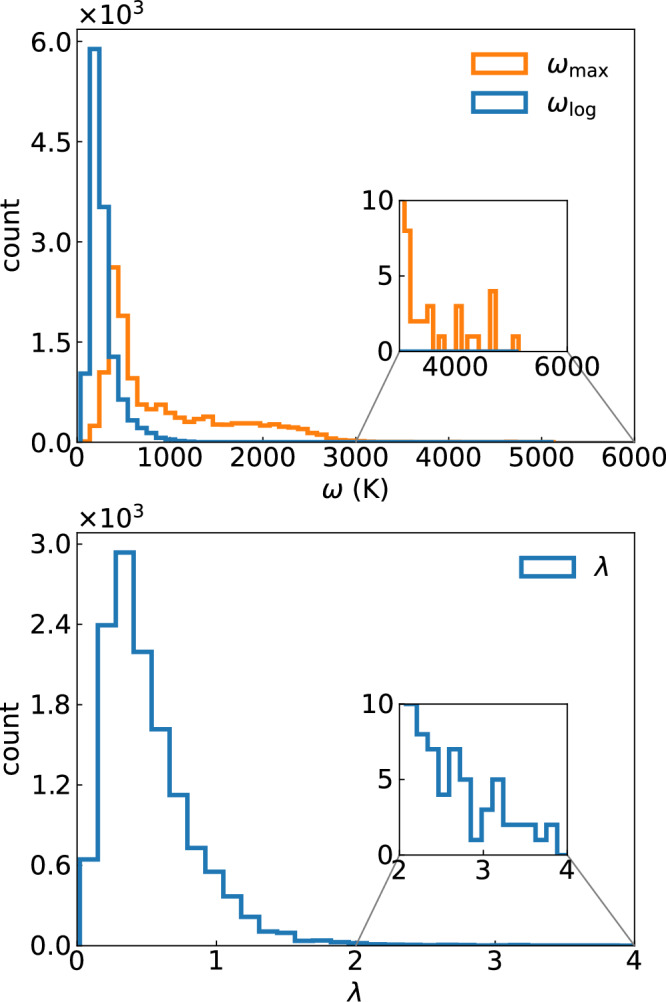
Histogram of el-ph coupling properties. Histogram of (top) the maxima (${\omega }_{\max }$) and logarithmic average (${\omega }_{\log }$) of phonon frequencies and (bottom) the electron-phonon coupling constants (*λ*) calculated for around 20,000 metals (see ref. ^43^ for computational details).

The same conclusion can be reached by looking at the calculated values for our materials depicted in Fig. 1. The distribution of values of ${\omega }_{\max }$ exhibits two maxima, one at around 500 K and another around 2000 K. Although the overwhelming majority of the compounds have ${\omega }_{\max }$ below 3000 K, we find a few that extend to 5400 K, well beyond the estimation of ref. ^42^. Not surprisingly, all of these are hydrides. An example of one such material, with a calculated ${\omega }_{\max }=5396$ K, is the hypothetical AgTl_2_H_2_. This is a compound that contains isolated H_2_ molecules inside a AgTl_2_ framework (see Supplementary Information, SI). The H–H distance is 0.78 Å, only slightly larger than the value of 0.76 Å in solid hydrogen calculated in the same approximation. As expected by the extreme difference of masses, the phonon band structure splits into separated manifolds, with the lowest lying states with Tl and Ag character and the highest states coming from H. The H_2_ stretching mode has negligible dispersion, and can be found at around 5400 K. The Ag–Tl and lowest lying H-modes all couple very strongly to the electrons, leading to the large *λ* = 1.1 (and a *T*_c_ ~ 11.5 K). As expected by the very high frequency, the highest lying phonon mode has a negligible contribution to *λ*, even if *α*^2^*F*(*ω*) exhibits a very high peak at that frequency.

In Fig. 1 we also plot the distribution of the values of ${\omega }_{\log }$. Contrary to ${\omega }_{\max }$, ${\omega }_{\log }$ has a single peak at very low frequency, and decays rapidly with frequency. At the end of the tail, at ${\omega }_{\log }$ values in excess of 1800 K we find a few hydrides, such as the hypothetical perovskite NaNiH_3_, and some ordered crystals of boron-doped sp^3^ carbon (see SI). In the case of the hydride, only the high frequency H-modes have a significant coupling to the electrons, leading to a large logarithmic average but a very small value of *λ* and consequently of *T*_c_. The boron-doped case takes advantage of the high-energy of the carbon modes (due to the very strong C–C sp^3^ bond) and from the fact that phonon modes couple strongly to the electrons in a large energy range. As expected, also in this case *λ* has moderate values, of the order of 0.5–0.6, leading to *T*_c_ in the range of 10–30 K.

As the determinant factor for the calculation of *T*_c_ is ${\omega }_{\log }$ and not ${\omega }_{\max }$, from this discussion it seems much more reasonable to use values of the order of 1800 K, and not ${\omega }_{\max }=\, 3680$ K, as in ref. ^42^.

The next step in the estimation of the maximum value of *T*_c_ is the optimization of the shape of *α*^2^*F*(*ω*), assuming a maximum phonon frequency of ${\omega }_{\max }$. A free optimization of this function would obviously lead to *T*_c_ = *∞*, as *α*^2^*F*(*ω*) is not constrained by any sum-rule. Therefore, Trachenko et al. fixed *λ* = 2^42^, the value that maximizes *T*_c_according to McMillan’s formula. From the considerations above, and from the lower panel of Fig. 1, *λ* = 2 seems to be a reasonable value, perfectly reachable in a variety of compounds. The hypothetical compounds we have in our dataset with highest values of *λ* ≈ 3.3 are ClB_2_C_8_ and Al_2_OsH_7_ (see SI). The former is a C clathrate p-doped with B and co-doped with endohedral Cl, with a ${\omega }_{\log }=425$ K and a calculated *T*_c_ = 55 K. The latter, that exhibits a Al_2_Os framework that includes a large quantity of hydrogen, has an ${\omega }_{\log }$ of almost 300 K, leading to *T*_c_ = 38 K.

The optimal shape of *α*^2^*F*(*ω*) obtained by Ref. ^42^ by optimizing the *T*_c_ calculated from the Eliashberg equations is a narrow peak at ${\omega }_{\max }$. Actually, the same conclusion follows directly from McMillan’s formula for *T*_*c*_. For a fixed value of *λ*, the value of *T*_c_ changes linearly with ${\omega }_{\log }$. In turn, the maximum value of ${\omega }_{\log }$ is obtained when ${\alpha }^{2}F(\omega ) \sim \delta (\omega -{\omega }_{\max })$, leading to ${\omega }_{\log }=\, {\omega }_{\max }$. If, for the sake of the argument, we insert *λ* = 2, ${\omega }_{\log }=1800$ K into the McMillan function, and assume a value of μ^* ^= 0.1, we obtain *T*_c_ = 260 K. This is smaller than the value of ref. ^42^, but still much larger than the current record of MgB_2_.

It is most likely, however, that this limit is unattainable for any physical system at ambient pressure. In fact, the parameters ${\omega }_{\log }$ and *λ* are not entirely independent, as they are two different moments of the same *α*^2^*F*(*ω*) function. This was already recognized in the seminal work of McMillan in 1968^36^, where it was shown that *λ* is inversely proportional to the square of the average phonon frequency. Obviously ${\omega }_{\log }$ is favored by high frequencies, while *λ* by low frequencies, so the shape of the optimal *α*^2^*F*(*ω*) that maximizes *T*_c_ (and that only contains a *single*, very high-frequency, flat-band phonon mode that couples very strongly to the electrons) seems to be unattainable physically.

**Fig. 2:**
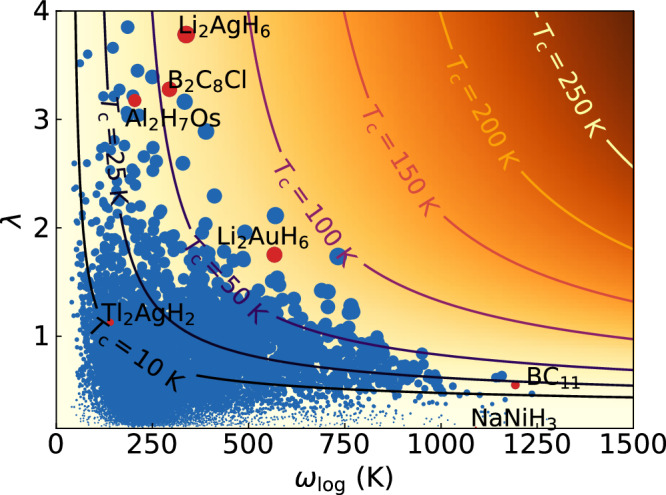
Scatter plot of ${\omega }_{\log }$ versus *λ* for all calculated systems. The size of the circles is proportional to *T*_c_ calculated from Allen-Dynes formula with correction factors^37^ and μ^*^ = 0.1. The contour lines are included as a guide to the eye and are obtained from McMillan’s formula of eq. (1) with μ^*^ = 0.1.

This reasoning is also supported by the data depicted in Fig. 2, where we plot the relationship between ${\omega }_{\log }$ and *λ* for all compounds in our dataset. The size of the circles is proportional to *T*_c_ and in the background we plot the contour lines of constant *T*_c_ as calculated from McMillan’s formula with μ^*^ = 0.1. We see that, as expected, compounds with very large values of ${\omega }_{\log }$ often have small *λ* and vice-versa. Furthermore, no material comes close to the optimal case, and compounds with the highest values of *T*_c_ are the ones that achieve a good compromise between reasonable high ${\omega }_{\log }$ and *λ*.

**Fig. 3:**
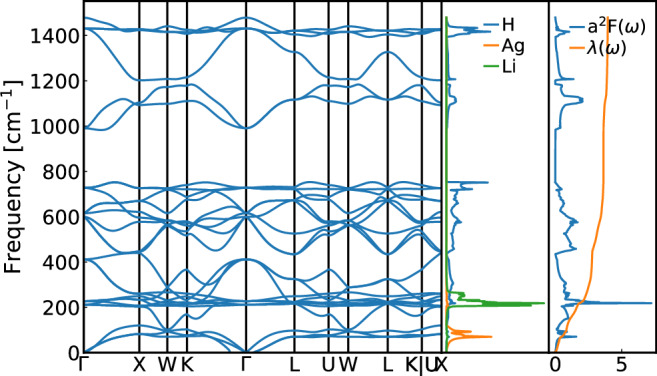
Phonon and electron phonon coupling spectra of Li_2_AgH_6_. From left to right, the phonon band structure, density of phonon states, and Eliashberg spectral function α^2^F(ω), calculated within the harmonic approximation.

As an example, we will look at the compounds with the highest *T*_c_ in our dataset, specifically Li_2_AgH_6_ and Li_2_AuH_6_ (see Fig. 3 and Fig. S1 in SI). These compound crystallize in the same cubic structure as Mg_2_IrH_6_, Mg_2_PdH_6_, Mg_2_PtH_6_, etc. that were recently proposed^85–87^ as high-*T*_c_ superconductors. Both materials are thermodynamically unstable at ambient pressure (see Fig. S2 in SI) at respectively 0.319 eV/atom and 0.172 eV/atom above the convex hull for the Ag and the Au compounds, and are not significantly stabilized by pressure (at least up to 50 GPa, see SI).

The electronic band structure of Li_2_AgH_6_ resembles the one of Mg_2_IrH_6_, with a single band crossing the Fermi surface. This band, with very strong H-character, is however more dispersive in the present case. Also the phonon band structure is similar, with the acoustic modes mostly composed by vibrations of the heavier atom, followed by modes stemming from the alkali or alkaline earth metal. Finally, there are three separate manifolds of phonon bands. In Li_2_AgH_6_ the lowest manifold contains both H and Li character due to the small difference between the atomic masses of these elements. Essentially all modes contribute to the very high value of *λ* ≈ 4.

We would like to note that one of the acoustic phonon branches exhibits (small) imaginary values close to the *Γ*. This is a shortcoming of the harmonic approximation, and the structure is perfectly dynamically stable when anharmonic and quantum nuclear effects are taken into account (see SI)^88,89^. In any case, 95% of the electron-phonon coupling comes from mid-low frequency modes which are almost identical between harmonic and anharmonic calculations.

In order to obtain a more accurate *T*_c_ estimation within modern superconductivity methods we have recomputed the value of *T*_c_ using two state-of-the art approaches which, unlike more conventional methods, include the electron phonon coupling and the Coulomb interaction calculated from first-principles. These are the full Eliashberg approach of Ref. ^90^ and density functional theory for superconductors^91–95^ (SCDFT). For the sake of these high-accuracy calculations the electron-phonon coupling was recomputed using a Monte-Carlo *k*-mesh accumulated on the Fermi surface, on which the electron-phonon matrix elements are linearly interpolated^96^. This ensures a perfect convergence of the nesting properties entering the definition of the Eliashberg spectral function.

The resulting critical temperature for Li_2_AgH_6_ is 108.8 K in Eliashberg theory and 83.0 K in SCDFT. Although both approaches are derived from the Migdal approximation for the electron-phonon self energy^97^ and assume a static Coulomb interaction, the slightly different *T*_c_ prediction is dictated by the approximation chain that leads to each computational scheme. On one hand, in SCDFT there is the approximation to the anomalous exchange-correlation functional^92^, which adds additional approximations to the form of the self-energy. On the other hand, in Eliashberg theory the energy dependence of the Coulomb interaction is neglected at the scale of the phononic energies. However, considering that the electron-phonon coupling parameter *λ* is very high, the functional approximation of SCDFT might be slightly beyond its validity range^92^. Therefore we expect that, in this case, the Eliashberg estimation should be more accurate.

It is interesting that the Eliashberg estimation of *T*_c_ is in very good agreement with the one obtained by means of the McMillan-Allen-Dynes approach using a standard μ^*^ = 0.1. This indicates that Coulomb interactions act as expected for a conventional sp-system^98^. This differs from what is observed in Mg_2_IrH_6_ which, as discussed in ref. ^85^, has its *T*_c_ overestimated by the μ* model^99^. The reason is the presence of a peak in the density of states at the Fermi level, that is close to a large band gap, leading to a poor Coulomb renormalization^98,100^. The Li_2_AgH_6_ system also features a peak in the density-of-states and a band gap, however the peak is broader, while the band gap is very small, leading to an overall smoother density profile and efficient Coulomb renormalization.

A similar analysis can be extended to Li_2_AuH_6_. This system has electronic, phononic and superconducting properties nearly identical to its Ag twin. The predicted superconducting *T*_c_ including Coulomb interactions is 91.0 K and 116.1 K in SCDFT and Eliashberg, respectively.

In view of the discussion before, these materials seem to be ideal cases for conventional superconductivity, and their *T*_c_ is likely in the maximum range of what can be achievable at ambient pressure. We note that these are isotropic superconductors, where the effect of anisotropy accounts for less than 1% of the value of *T*_c_. This is the opposite of MgB_2_, where the electron-phonon coupling mostly acts on the *σ* bands, and an isotropic calculations leads to an underestimation of *T*_c_ by almost a factor of two. The isotropic superconducting state of Li_2_AgH_6_ or Li_2_AuH_6_, if experimentally realized, would not only have a critical temperature above liquid nitrogen, but it would also be more suitable for high-field applications. In fact these applications require impurities and defects to work as pinning centers for magnetic flux lines and reduce the Ginsburg-Landau coherence-length. Isotropic superconductors are more likely to be stable upon the introduction of crystalline defects and still be affected by their scattering potential.

At this point it it important to recognize the approximations used in our workflow, and discuss their potential impact on the maximum value of *T*_c_.

### Finite size of the dataset

By using our machine learning model we have by now screened more than 100 million compounds, including almost 3 million hydrides. For all compounds predicted by the machine learning model to have a *T*_c_ above 10 K, we have performed full electron-phonon calculations. We also performed successive cycles of training the model and predicting *T*_*c*_, creating an iterative refinement process that has exhaustively explored the chemical space. By now, the error of our machine learning model has converged to below 1 K, indicating that we have captured the essential physics of electron-phonon coupling in these materials. Furthermore, all our recent batches consistently fail to find any new superconductors with very large *T*_*c*_ (>50 K), even as our material database grows at an impressive rate of 1–2 million compounds per week. This plateau in discoveries, despite exponentially increasing data, provides strong statistical evidence that we have identified the fundamental upper limits of superconducting transition temperatures.

### Anisotropy and multi-band effects

We have been systematically performing anisotropic calculations, either by solving the anisotropic Eliashberg equations or through density-functional theory for superconductors. For all compounds that appear at the top of our *T*_c_ list, anisotropic effects turn out to be very minor, and do not affect *T*_*c*_ significantly. This points to the conclusion that our best compounds are already “fully optimized”, and *T*_c_ has indeed reached its maximum value. Of course, for compounds with a lower transition temperature there is a larger margin for optimization. This can be seen from, e.g., ref. ^101^, where anisotropic calculations were performed for 242 materials, finding a significant increase in the *T*_c_ for a few compounds. The resulting *T*_c_ values, however, are still well below our suggested maximum *T*_c_ of around 100 K, in agreement with our arguments.

### Doping

Although this is a crucial strategy to improve superconducting properties we evaluate that it will not significantly affect our statistical analysis of the maximum *T*_c_. Let us divide this discussion in two parts: a) standard doping, where a small percentage of dopant atoms is added, removed or substituted, in an attempt to move the Fermi level to a region of higher density of states or of softening some phonons to a sweet spot in frequency. In a first approximation this can be achieved by a rigid shift of the Fermi energy in the calculations. We have tried this approach for our best candidates with partial success (see, e.g., Mg_2_PdH_6_ and Mg_2_PtH_6_^85^). In fact, materials with high critical temperature are usually already in a near-optimal configuration, and in this case their *T*_c_ can not be significantly improved by doping. This is the case of MgB_2_^102^ or Mg_2_IrH_6_^85^, for example. Of course, other compounds with lower values of *T*_c_ have a larger potential for optimization, but their optimized *T*_c_ remains moderately low. b) degenerate doping, where a significant amount of atoms is added, removed or substituted. Indeed this often leads to substantial increase of *T*_c_, as it can be seen, for example, in Ref. ^69^. However, this kind of systems are to a large extent already represented in our dataset, and should therefore be easily predicted by our machine-learning model. While our analysis focuses on ordered crystalline compounds, we note that the effect of alloying or disorder in doped systems remains a challenging computational problem that has been addressed in only a limited number of studies^103,104^.

### Anharmonic effects

Phonon anharmonic effects can be crucial for many systems. However, their primary impact lies in the stabilization of otherwise unstable or marginally stable phases. This stabilization mechanism is exemplified in H_3_S, where anharmonic effects enable the cubic phase to become stable at lower pressures than predicted by the harmonic approximation^105^. Furthermore, the stabilization of soft-modes usually leads to a decrease of *λ* and consequent decrease of *T*_c_ as, for example, in H_3_S under high pressure^105^ or in PdH at ambient pressure^106^.

### Reduced dimensionality

Recently some of us applied our approach to 2D systems^107^, motivated by the hypothesis that reduced dimensionality could enhance transition temperatures. However, the values of *T*_*c*_ for 2D systems turned out to be systematically lower than for 3D compounds. In retrospect, this outcome can be understood through fundamental physical principles. First, 2D systems exhibit systematically lower electronic densities of states at the Fermi level compared to 3D materials. Second, the frequencies of out-of-plane phonon modes are characteristically reduced in layered structures. While this frequency reduction does lead to an increase in the electron-phonon coupling parameter *λ*, it simultaneously causes a substantial decrease in the logarithmically averaged phonon frequency ${\omega }_{\log }$. According to the McMillan formula, the net effect of these competing factors results in an overall reduction of *T*_c_, despite the enhanced coupling strength.

### Treatment of the Coulomb interaction

**Fig. 4:**
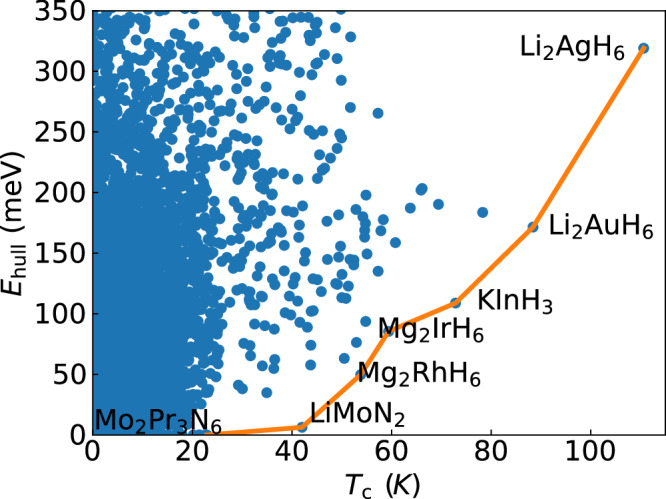
Scatter plot of the distance to the convex hull of stability versus the superconducting transition temperature for all compounds in our dataset. In orange we also indicate the Pareto front corresponding to this data. Compounds on the Pareto front are labeled. Values of *T*_c_ care alculated from Allen-Dynes formula with correction factors^37^ and μ* = 0.1.

Most often the Coulomb interaction is handled at the level of μ* approximation, and this is also the case for the data in Figs. 2 and 4. The standard value of μ* = 0.1 (that we also adopt) is likely a lower limit for real systems, and it is often a gross underestimation for compounds with very high values of *T*_c_. For example, for Mg_2_IrH_6_^85^ we computed the Coulomb interaction from first principles, and found that the resulting repulsion is extremely large at the Fermi level (yielding a *μ* = 0.58), consistent with the large electronic density of states. Furthermore, as the density of states decreases away from the Fermi level, the Coulomb renormalization mechanism becomes very inefficient. A direct calculation of μ* is quite difficult, but a rough estimation indicates that the value should be μ* ≫ 0.25, much larger than the 0.1 value commonly used, hence leading to a considerable decrease of *T*_c_.

Finally, we would like to discuss the issue of synthesizeability. Most of the compounds discussed above, and others that have been proposed in the literature, with high-*T*_c_ are hypothetical, and up to now none has been stabilized at ambient pressure. To better understand this problem, we plot in Fig. 4 the distance to the convex hull of thermodynamic stability and the calculated transition temperature of all compounds in our dataset. We also indicate the Pareto front that corresponds to this data. The values of *T*_c_ are obtained with an isotropic theory, so the transition temperature of MgB_2_ is considerably underestimated. We also note that although being close to the hull is not a synonym of synthesizeability, the higher the distance to the hull for a given compound, the smaller the probability that this compound can be stabilized experimentally.

Very close to the hull, we find that the compound with highest predicted *T*_c_ is LiMoN_2_ with around 40 K^44^. Unfortunately, this compound exhibits intrinsic defects that lower the high density of states at the Fermi level and destroy superconductivity^44,108^. If it is possible to resolve this problem experimentally and synthesize the pristine compound is at this point unknown^109^. At higher values of *T*_c_ we find several hydrides and boron-carbides. Unfortunately, all these compounds are unstable thermodynamically with a distance to the hull that increases rapidly along the Pareto front. This is easy to understand as hydrides prefer charge-compensated, semiconducting (or insulating) phases and are therefore destabilized in the metallic phase, and boron induces stresses in the very strong diamond framework increasing its energy. A possible path through the high-pressure synthesis followed by quenching of these compounds to ambient pressure is commonly suggested in the literature^110–112^. This approach was recently used to quench a superconducting phase of the topological alloy compound Bi_0.5_Sb_1.5_Te_3_^113^ from 4 GPa to ambient pressure with *T*_*c*_ reaching 10 K. Also FeSe has been subject to pressure quenching from 4.15 GPa reaching a *T*_*c*_ of 37 K at ambient pressure^114^, as well as Sb, quenched from 10.9 GPa and retaining a *T*_*c*_ of 3 K at ambient pressure. Another example is the clathrate SrB_3_C_3_, synthesized under high pressure and measured to be a superconductor with a maximum *T*_*c*_ of 22 K at 23 GPa^68^. In the latter case, it was argued that the sample survived to ambient pressure, but it was not possible to measure superconductivity. Despite these successes, the possibility of quenching superconducting hydrides (with extremely mobile hydrogen atoms) from the very high pressures required for their synthesis to ambient conditions is still unproved to this date, and remains highly speculative.

In summary, by analyzing data from more than 20,000 electron-phonon calculations we critically discussed the possible maximum *T*_c_ of conventional superconductors at ambient pressure. It seems clear that it is possible to design hypothetical compound with values of *T*_c_ reaching 100–120 K, much larger than the current experimental record, but still very far from room temperature. Unfortunately, all compounds with high *T*_c_ appear to be thermodynamically unstable, raising questions about their experimental synthesis and characterization. It is true that physical laws do not restrict *T*_c_ to go beyond 100–120 K, but in practice our data show that the experimental realization of a compound with such high *T*_c_ is extremely unlikely.

However, we do not exclude that room-temperature superconductivity could be achieved through unconventional pairing mechanisms, high pressure conditions, or entirely new physical phenomena that transcend current theoretical frameworks. Our hope is that this analysis helps guide future research toward the most promising avenues while establishing realistic expectations about the fundamental limits of conventional electron-phonon mediated superconductivity.

## Methods

Density-functional calculations were executed using version 6.8 and 7.1 of QUANTUM ESPRESSO^115,116^ with the Perdew-Burke-Ernzerhof functional for solids (PBEsol)^117^ generalized gradient approximation. For pseudopotentials we used the stringent, scalar-relativistic norm-conserving PBEsol set from PSEUDODOJO project^118^. Geometry optimizations were conducted using a uniform *Γ*-centered *k*-point grid with a density of 1500 k-points per reciprocal atom. Convergence thresholds for energies, forces, and stresses were established at 1 × 10^−8^ a.u., 1 × 10^−6^ a.u., and 5 × 10^−2^ kbar, respectively. For the electron-phonon coupling calculations, we implemented a double-grid technique, utilizing a 3000 k-points per recip- rocal atom *k*-grid as the coarse grid, and a fine grid with 6000 k-points per reciprocal atom. For the *q*-sampling of phonons, we employed a *q*-point grid with half the density of the coarse *k-*grid. The double *δ*-integration to obtain the Eliashberg function was performed with a Methfessel-Paxton smearing of 0.05 Ry. *T*_c_ were estimated with the Allen-Dynes formula with correction factors^37^.

## Supplementary information

Supplementary Information

Transparent Peer Review file

## Data Availability

The data generated in this study have been deposited in the Alexandria database at https://alexandria.icams.rub.de/.

## Code availability

All codes are publicly available on GitHub at https://github.com/hyllios/utils/^119^.
