# Supplementary material for: The maximum Tc of conventional superconductors at ambient pressure
Source: Nat Commun. 2025 Sep 10;16:8253. doi: 10.1038/s41467-025-63702-w (PMC12423309; doi:10.1038/s41467-025-63702-w)
Supplement: Supplementary file 1 — Supplementary Information [file 41467_2025_63702_MOESM1_ESM.pdf]

Supplementary Information for: The Maximum T_c of Conventional Superconductors at Ambient Pressure

Kun Gao,[1] Tiago F. T. Cerqueira,[2] Antonio Sanna,[3] Yue-Wen Fang,[4] Đorđe Dangić,[4,5] Ion Errea,[4,5,6] Hai-Chen Wang,[1] Silvana Botti,[1] and Miguel A. L. Marques[1,*]

[1]*Research Center Future Energy Materials and Systems of the University Alliance Ruhr and Interdisciplinary Centre for Advanced Materials Simulation, Ruhr University Bochum, Universitätsstraße 150, D-44801 Bochum, Germany*
[2]*CFisUC, Department of Physics, University of Coimbra, Rua Larga, 3004-516 Coimbra, Portugal*
[3]*Max-Planck-Institut für Mikrostrukturphysik, Weinberg 2, D-06120 Halle, Germany*
[4]*Centro de Física de Materiales (CFM-MPC), CSIC-UPV/EHU, Manuel de Lardizabal Pasealekua 5, 20018 Donostia/San Sebastián, Spain*
[5]*Fisika Aplikatua Saila, University of the Basque Country (UPV/EHU), Europa Plaza 1, 20018 Donostia/San Sebastián, Spain*
[6]*Donostia International Physics Center (DIPC), Manuel de Lardizabal Pasealekua 4, 20018 Donostia/San Sebastián, Spain*

(Dated: August 20, 2025)

* miguel.marques@rub.de

#1: Tl_2AgH_2

**mat id**	agm002924843
**spg**	139
**nsites**	5
**e above hull**	0.124 eV
**e form**	0.101 eV
**decomposition**	Tl, Ag, TlH_4
**ecutwfc**	100.0 Ry
**kpts coarse**	16×16×16
**kpts fine**	32×32×32
**qpts**	4×4×4
λ	1.128
$\omega_{\mathbf{log}}$	141 K
ω_2	475 K
$T_{\mathbf{c}}^{\mathbf{Mcmillan}}$	11.7 K
$T_{\mathbf{c}}^{\mathbf{Allen\text{-}Dynes}}$	12.7 K
$T_{\mathbf{c}}^{\mathbf{Eliashberg}}$	13.2 K

Primitive structure:
a: 5.0017 Å, b: 5.0017 Å, c: 5.0017 Å
α: 118.36°, β: 118.36°, γ: 92.87°

Tl	[0.7500, 0.2500, 0.5000]
Tl	[0.2500, 0.7500, 0.5000]
Ag	[0.0000, 0.0000, 0.0000]
H	[0.5555, 0.5555, 0.0000]
H	[0.4445, 0.4445, 0.0000]

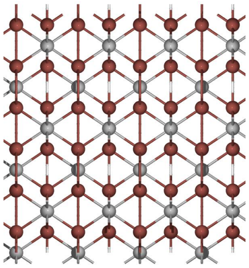
from a-axis

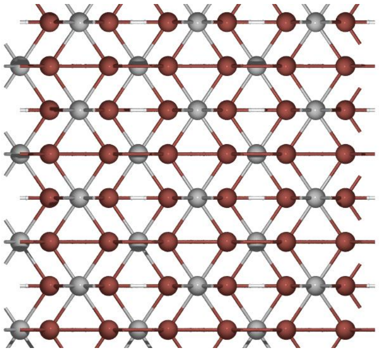
from b-axis

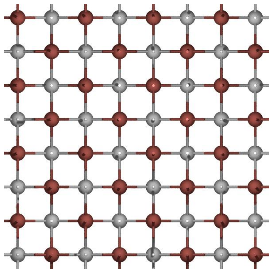
from c-axis

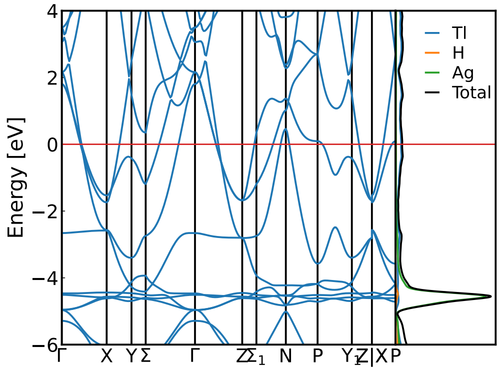

Electron band structure

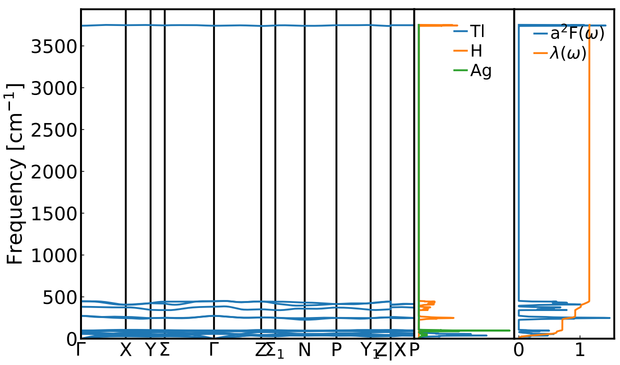

Phonon band structure

#2: $NaNiH_3$

**mat id**	agm002490730
**spg**	221
**nsites**	5
**e above hull**	0.014 eV
**e form**	-0.241 eV
**decomposition**	$NaNiH_3$
**ecutwfc**	116.0 Ry
**kpts coarse**	16×16×16
**kpts fine**	32×32×32
**qpts**	4×4×4
λ	0.158
$\omega_{\mathbf{log}}$	1090 K
ω_2	1420 K
$T_{\mathbf{c}}^{\mathbf{Mcmillan}}$	0.0 K
$T_{\mathbf{c}}^{\mathbf{Allen\text{-}Dynes}}$	0.0 K
$T_{\mathbf{c}}^{\mathbf{Eliashberg}}$	0.0 K

Primitive structure:

a: 3.3957 Å, b: 3.3957 Å, c: 3.3957 Å

α: 90.00°, β: 90.00°, γ: 90.00°

Na	[0.5000, 0.5000, 0.5000]
Ni	[0.0000, 0.0000, 0.0000]
H	[0.0000, 0.5000, 0.0000]
H	[0.0000, 0.0000, 0.5000]
H	[0.5000, 0.0000, 0.0000]

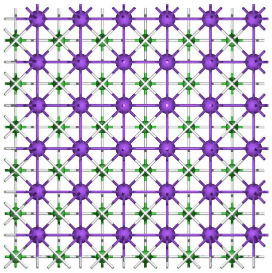

from a-axis

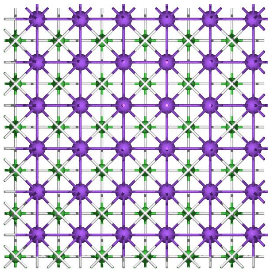

from b-axis

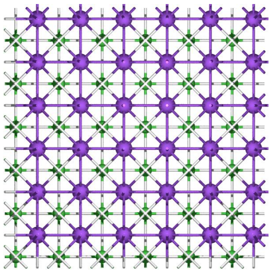

from c-axis

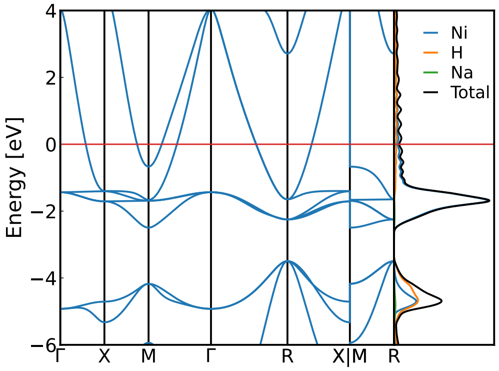

Electron band structure

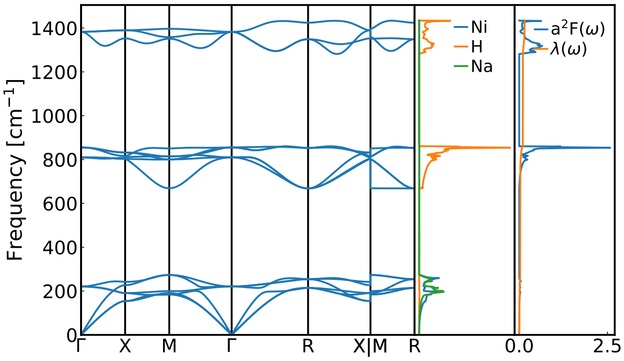

Phonon band structure

#3: BC_{11}

**mat id**	agm006148322
**spg**	21
**nsites**	12
**e above hull**	0.225 eV
**e form**	0.214 eV
**decomposition**	C, B_4C
**ecutwfc**	90.0 Ry
**kpts coarse**	12×12×16
**kpts fine**	24×24×32
**qpts**	3×3×4
λ	0.551
$\omega_{\mathbf{log}}$	1193 K
ω_2	1269 K
$T_{\mathbf{c}}^{\mathbf{Mcmillan}}$	20.7 K
$T_{\mathbf{c}}^{\mathbf{Allen\text{-}Dynes}}$	21.2 K
$T_{\mathbf{c}}^{\mathbf{Eliashberg}}$	24.0 K

Primitive structure:

a: 5.7150 Å, b: 5.7150 Å, c: 3.5998 Å

α: 90.00°, β: 90.00°, γ: 143.32°

B [0.0000, 1.0000, 0.5000]
C [0.1696, 0.3409, 0.7555]
C [0.3332, 0.6668, 0.5000]
C [0.5000, 1.0000, 0.7471]
C [0.1673, 0.8327, 0.0000]
C [0.3409, 0.1696, 0.2445]
C [0.6668, 0.3332, 0.5000]
C [0.8304, 0.6591, 0.7555]
C [0.5000, 0.5000, 0.0000]
C [0.6591, 0.8304, 0.2445]
C [0.8327, 0.1673, 0.0000]
C [0.0000, 0.5000, 0.2529]

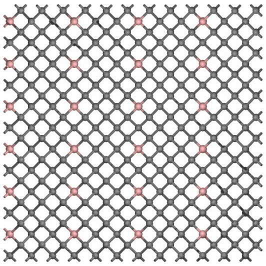

from a-axis

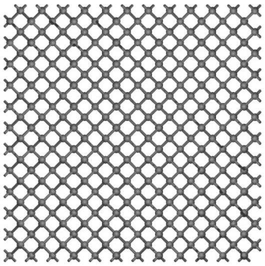

from b-axis

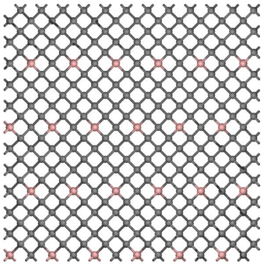

from c-axis

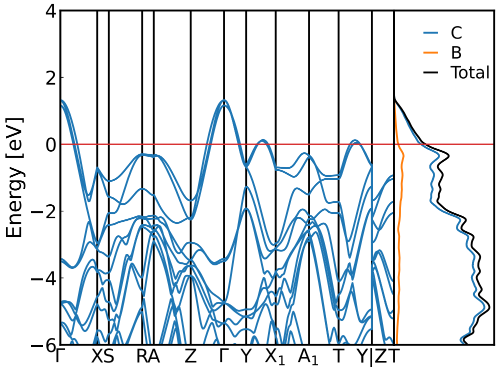

Electron band structure

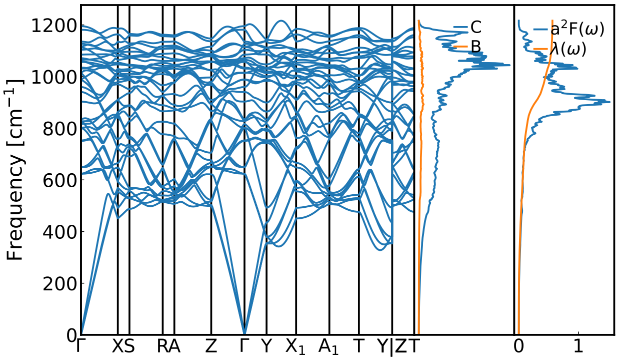

Phonon band structure

#4: B_2C_8Cl

**mat id**	agm029547244
**spg**	225
**nsites**	11
**e above hull**	0.989 eV
**e form**	0.798 eV
**decomposition**	B_4C, BCl_3, C
**ecutwfc**	90.0 Ry
**kpts coarse**	12×12×12
**kpts fine**	24×24×24
**qpts**	3×3×3
λ	3.278
$\omega_{\mathbf{log}}$	294 K
ω_2	492 K
$T_{\mathbf{c}}^{\mathbf{Mcmillan}}$	55.0 K
$T_{\mathbf{c}}^{\mathbf{Allen\text{-}Dynes}}$	82.7 K
$T_{\mathbf{c}}^{\mathbf{Eliashberg}}$	96.1 K

Primitive structure:

a: 4.9498 Å, b: 4.9498 Å, c: 4.9498 Å
α: 60.00°, β: 60.00°, γ: 60.00°

B [0.2500, 0.2500, 0.2500]
B [0.7500, 0.7500, 0.7500]
C [0.8879, 0.3364, 0.8879]
C [0.1121, 0.6636, 0.1121]
C [0.8879, 0.8879, 0.8879]
C [0.1121, 0.1121, 0.1121]
C [0.3364, 0.8879, 0.8879]
C [0.6636, 0.1121, 0.1121]
C [0.8879, 0.8879, 0.3364]
C [0.1121, 0.1121, 0.6636]
Cl [0.5000, 0.5000, 0.5000]

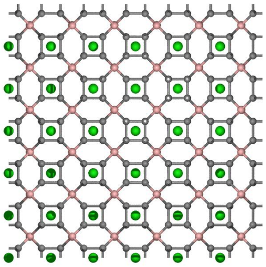

from a-axis

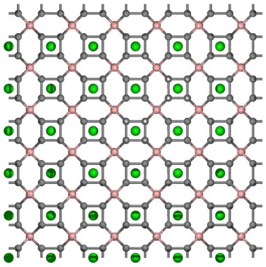

from b-axis

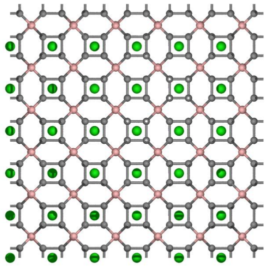

from c-axis

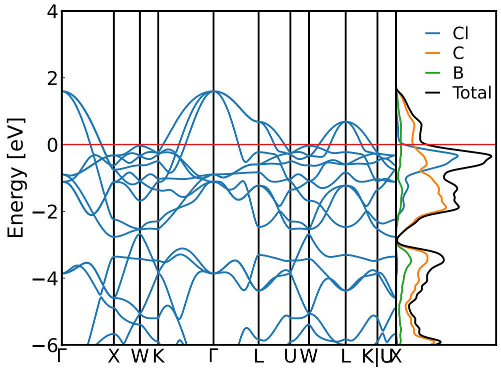

Electron band structure

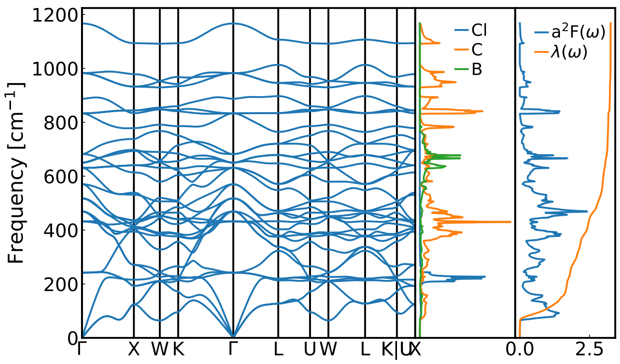

Phonon band structure

#5: Al_2H_7Os

**mat id**	agm006206343
**spg**	225
**nsites**	10
**e above hull**	0.265 eV
**e form**	0.097 eV
**decomposition**	Al_2Os, H_2
**ecutwfc**	86.0 Ry
**kpts coarse**	12×12×12
**kpts fine**	24×24×24
**qpts**	3×3×3
λ	3.175
$\omega_{\mathbf{log}}$	204 K
ω_2	596 K
$T_\mathbf{c}^{\mathbf{Mcmillan}}$	37.6 K
$T_\mathbf{c}^{\mathbf{Allen\text{-}Dynes}}$	57.3 K
$T_\mathbf{c}^{\mathbf{Eliashberg}}$	100.0 K

Primitive structure:

a: 4.5896 Å, b: 4.5896 Å, c: 4.5896 Å
α: 60.00°, β: 60.00°, γ: 60.00°

Al	[0.7500, 0.7500, 0.7500]
Al	[0.2500, 0.2500, 0.2500]
H	[0.5000, 0.5000, 0.5000]
H	[0.2721, 0.2721, 0.7279]
H	[0.2721, 0.7279, 0.2721]
H	[0.7279, 0.2721, 0.7279]
H	[0.2721, 0.7279, 0.7279]
H	[0.7279, 0.2721, 0.2721]
H	[0.7279, 0.7279, 0.2721]
Os	[0.0000, 0.0000, 0.0000]

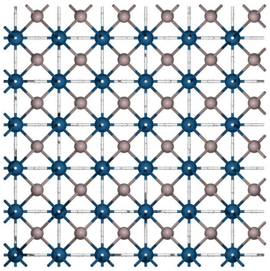
from a-axis

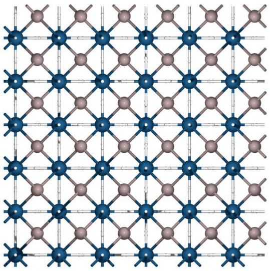
from b-axis

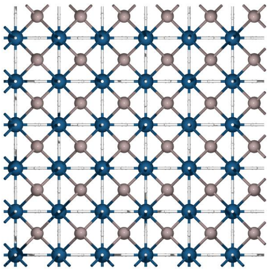
from c-axis

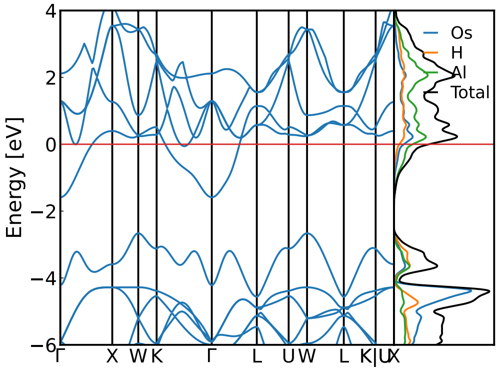

Electron band structure

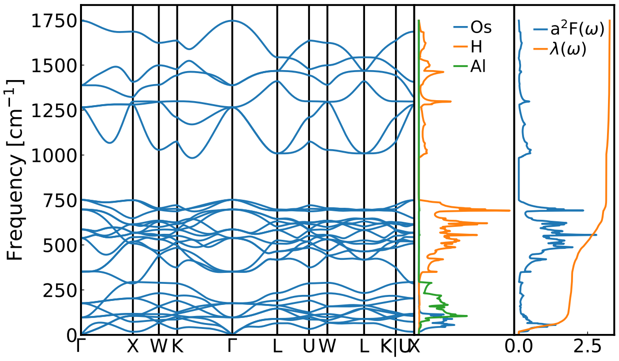

Phonon band structure

#6: Li_2AgH_6

**mat id**	agm006188332
**spg**	225
**nsites**	9
**e above hull**	0.319 eV
**e form**	0.094 eV
**decomposition**	LiH_9, Ag, LiH
**ecutwfc**	104.0 Ry
**kpts coarse**	12×12×12
**kpts fine**	24×24×24
**qpts**	3×3×3
λ	3.783
$\omega_{\mathbf{log}}$	338 K
ω_2	655 K
$T_{\mathbf{c}}^{\mathbf{Mcmillan}}$	66.5 K
$T_{\mathbf{c}}^{\mathbf{Allen\text{-}Dynes}}$	110.6 K
$T_{\mathbf{c}}^{\mathbf{Eliashberg}}$	132.3 K

Primitive structure:
a: 4.6213 Å, b: 4.6213 Å, c: 4.6213 Å
α: 60.00°, β: 60.00°, γ: 60.00°

Li	[0.7500, 0.7500, 0.7500]
Li	[0.2500, 0.2500, 0.2500]
Ag	[0.0000, 0.0000, 0.0000]
H	[0.2654, 0.2654, 0.7346]
H	[0.2654, 0.7346, 0.2654]
H	[0.7346, 0.2654, 0.7346]
H	[0.7346, 0.7346, 0.2654]
H	[0.2654, 0.7346, 0.7346]
H	[0.7346, 0.2654, 0.2654]

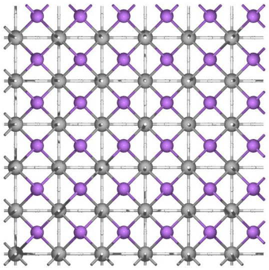
from a-axis

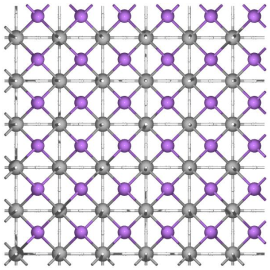
from b-axis

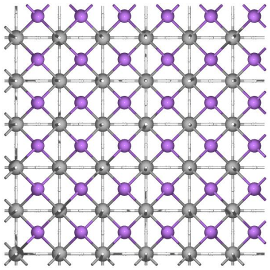
from c-axis

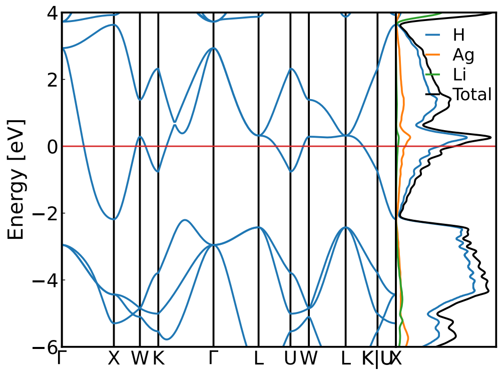

Electron band structure

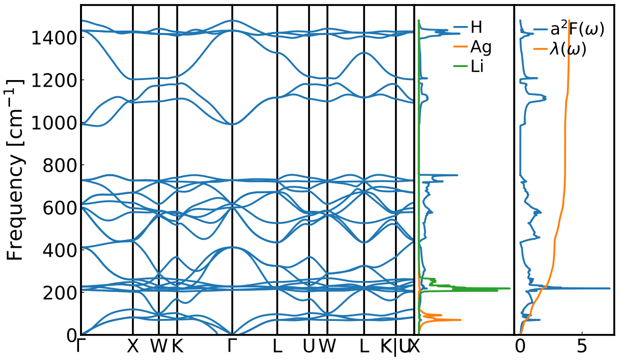

Phonon band structure

Density-functional calculations were executed using version 6.8 and 7.1 of QUANTUM ESPRESSO [1, 2] with the Perdew-Burke-Ernzerhof functional for solids (PBEsol) [3] generalized gradient approximation. For pseudopotentials we used the stringent, scalar-relativistic norm-conserving PBEsol set from PSEUDODOJO project [4]. Geometry optimizations were conducted using a uniform Γ-centered k-point grid with a density of 1500 k-points per reciprocal atom. Convergence thresholds for energies, forces, and stresses were established at 1×10^{-8} a.u., 1×10^{-6} a.u., and 5×10^{-2} kbar, respectively. For the electron-phonon coupling calculations, we implemented a double-grid technique, utilizing a $8 \times 8 \times 8$ k-grid as the coarse grid, and a $16 \times 16 \times 16$ as the fine grid. For the q-sampling of phonons, we employed a $2 \times 2 \times 2$ q-point grid. The double δ-integration to obtain the Eliashberg function was performed with a Methfessel–Paxton smearing of 0.05 Ry.

T_c were estimated with the Allen-Dynes formula with correction factors [5]:

$$T_c^{AD} = f_1 f_2 \frac{w_{\log}}{1.20} \exp\left[-1.04 \frac{1+\lambda}{\lambda - \mu^*(1+0.62\lambda)}\right] , \tag{1}$$

where

$$f_1 = \left\{1 + \left[\frac{\lambda}{2.46(1+3.8*\mu^*)}\right]^{3/2}\right\}^{1/3} , \tag{2a}$$

$$f_2 = 1 + \frac{\lambda^2(\omega_2/\omega_{\log} - 1)}{\lambda^2 + [1.82(1+6.3\mu^*)\omega_2/\omega_{\log}]^2} . \tag{2b}$$

To address the anharmonic phonon properties of Li_2AgH_6 and Li_2AuH_6 at ambient pressure, we used the stochastic self-consistent harmonic approximation (SSCHA) method [6–9]. In the case of Li_2AgH_6, a 4×4×4 supercell including 576 atoms was used in SSCHA calculations, which corresponds to the dynamical matrices on a commensurate 4×4×4 **q**-mesh. All the degrees of freedom were fully relaxed in the SSCHA calculations. Due to the heavy DFT calculations and the demanding number of structure configurations used for the convergence of Hessian phonons, we trained a Gaussian approximation potential [10] based on the DFT calculations. The converged free energy Hessian phonons were obtained by using the Gaussian approximation potential. In the anharmonic phonon calculations of Li_2AuH_6, we employed $2 \times 2 \times 2$ supercell with 72 atoms that is sufficient to get convergence. The cutoff energy was 80 Ry with $10 \times 10 \times 10$ **k**-point grid for the supercell calculations. We also performed electron-phonon coupling calculations for the anharmonic structure of Li_2AuH_6, on $6 \times 6 \times 6$ **q**-point grid with coarse **k**-point sampling of $24 \times 24 \times 24$ and dense grid of $42 \times 42 \times 42$. The double delta sum was done with 0.004 Ry Gaussian smearing. The results are summarized in Table S1.

TABLE S1. Calculated electron-phonon coupling constant, $\omega_{\log}$, ω_2, and T_c for Li_2AuH_6. The superconducting critical temperature is calculated with McMillan's formula and Allen-Dynes modified formula (AD) with $\mu^* = 0.1$, with harmonic phonons and anharmonic phonons from the SSCHA auxiliary force constants and those from the SSCHA free energy Hessian.

$\mu^* = 0.1$	Harmonic	Auxiliary	Hessian
λ	3.86	2.10	2.67
$\omega_{\log}$ (K)	317	572	503
ω_2 (K)	724	982	870
T_C(K) - McMillan	63	85	86
T_C(K) - AD	108	107	118

Fig. S1 shows the comparison between the harmonic phonons from DFPT, anharmonic free energy Hessian phonon spectra, and the anharmonic auxiliary phonon spectra for both Li_2AgH_6 and Li_2AuH_6.

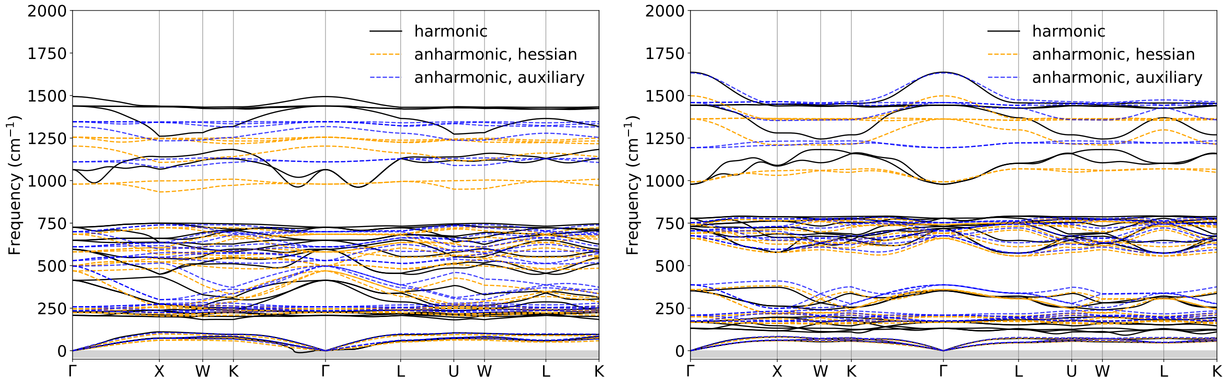

FIG. S1. Anharmonic free energy Hessian phonon spectra and anharmonic auxiliary phonon spectra of Li_2AgH_6 (left panel) and Li_2AuH_6 (right panel) calculated by the stochastic self-consistent harmonic approximation (SSCHA) at ambient pressure (the reader is referred to Refs. [7, 9] for details). The results are compared to the harmonic result. In the case of Li_2AgH_6 anharmonicity lifts the instability observed close to Γ in the harmonic case.

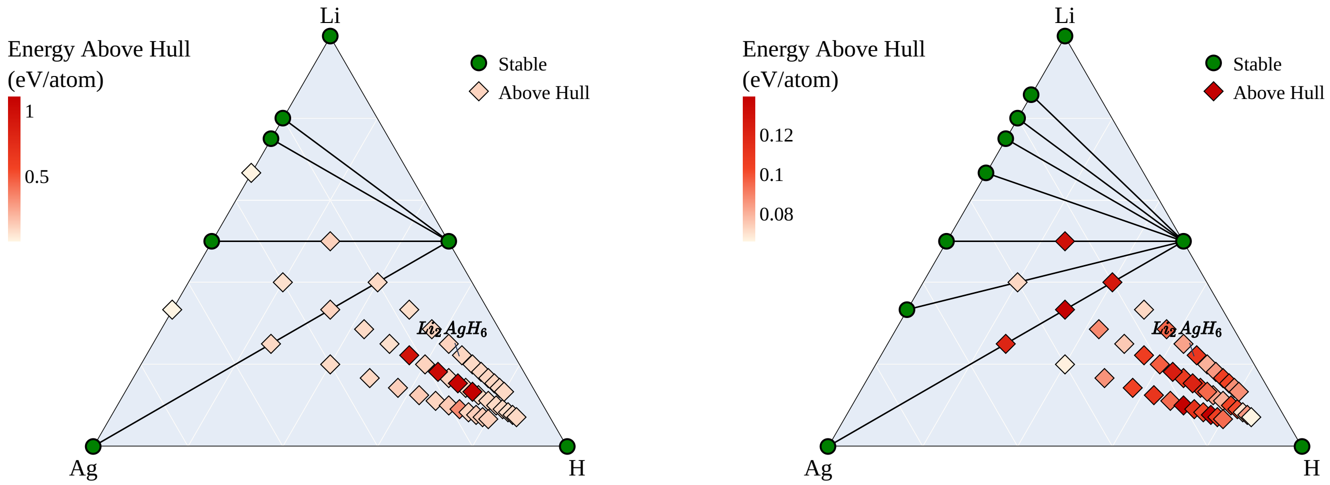

FIG. S2. The convex hull of the Li–Ag–H system at 25 GPa (left) and 50 GPa (right). Circles represent thermodynamically stable structures, and diamonds represent metastable structures.

SUPPLEMENTARY REFERENCES

[1] Giannozzi, P. *et al.* Quantum espresso: a modular and open-source software project for quantum simulations of materials. *J. Phys.: Condens. Matter* **21**, 395502 (2009). URL `http://dx.doi.org/10.1088/0953-8984/21/39/395502`.

[2] Giannozzi, P. *et al.* Advanced capabilities for materials modelling with quantum espresso. *J. Phys.: Condens. Matter* **29**, 465901 (2017). URL `http://dx.doi.org/10.1088/1361-648X/aa8f79`.

[3] Perdew, J. P. *et al.* Restoring the density-gradient expansion for exchange in solids and surfaces. *Phys. Rev. Lett.* **100** (2008). URL `http://dx.doi.org/10.1103/PhysRevLett.100.136406`.

[4] van Setten, M. *et al.* The pseudodojo: Training and grading a 85 element optimized norm-conserving pseudopotential table. *Comput. Phys. Commun.* **226**, 39–54 (2018). URL `http://dx.doi.org/10.1016/j.cpc.2018.01.012`.

[5] Allen, P. B. & Dynes, R. C. Transition temperature of strong-coupled superconductors reanalyzed. *Phys. Rev. B* **12**, 905–922 (1975). URL `https://link.aps.org/doi/10.1103/PhysRevB.12.905`.

[6] Errea, I., Calandra, M. & Mauri, F. Anharmonic free energies and phonon dispersions from the stochastic self-consistent harmonic approximation: Application to platinum and palladium hydrides. *Phys. Rev. B* **89**, 064302 (2014). URL `https://link.aps.org/doi/10.1103/PhysRevB.89.064302`.

[7] Bianco, R., Errea, I., Paulatto, L., Calandra, M. & Mauri, F. Second-order structural phase transitions, free energy curvature, and temperature-dependent anharmonic phonons in the self-consistent harmonic approximation: Theory and

stochastic implementation. *Phys. Rev. B* **96**, 014111 (2017). URL https://link.aps.org/doi/10.1103/PhysRevB.96.014111.

[8] Monacelli, L., Errea, I., Calandra, M. & Mauri, F. Pressure and stress tensor of complex anharmonic crystals within the stochastic self-consistent harmonic approximation. *Phys. Rev. B* **98**, 024106 (2018). URL https://link.aps.org/doi/10.1103/PhysRevB.98.024106.

[9] Monacelli, L. *et al.* The stochastic self-consistent harmonic approximation: calculating vibrational properties of materials with full quantum and anharmonic effects. *J. Phys. Condens. Matter.* **33**, 363001 (2021). URL https://doi.org/10.1088/1361-648x/ac066b.

[10] Bartók, A. P., Payne, M. C., Kondor, R. & Csányi, G. Gaussian approximation potentials: The accuracy of quantum mechanics, without the electrons. *Phys. Rev. Lett.* **104**, 136403 (2010). URL https://link.aps.org/doi/10.1103/PhysRevLett.104.136403.
