# Supplementary material for: The maximum Tc of conventional superconductors at ambient pressure
Source: Nat Commun. 2025 Sep 10;16:8253. doi: 10.1038/s41467-025-63702-w (PMC12423309; doi:10.1038/s41467-025-63702-w)
Supplement: Supplementary file 2 — Transparent Peer Review file [file 41467_2025_63702_MOESM2_ESM.pdf]

nature portfolio

Peer Review File

The Maximum T_C of Conventional Superconductors at Ambient Pressure

Corresponding Author: Dr Haichen Wang

This file contains all reviewer reports in order by version, followed by all author rebuttals in order by version.

Version 0:

Reviewer comments:

Reviewer #1

(Remarks to the Author)
The study leverages a dataset of over 20,000 metals to statistically analyze the interplay between electron-phonon coupling (λ) and logarithmic average phonon frequencies (ωlog). This provides a rare empirical foundation for estimating the upper limit of Tc in conventional superconductors. The prior assumptions (e.g., McMillan's formula) by demonstrating that the optimal α2F(ω) function for maximizing Tc is unphysical due to the inherent trade-off between ωlog and λ. This refines the understanding of phonon-mediated superconductivity. While the work provides valuable insights into conventional superconductivity, its exclusion of σ-electron systems, insufficient synthesis strategies, and dataset limitations significantly weaken the novelty and impact. These issues, compounded by the speculative nature of proposed materials, render the manuscript not suitable for publication in Nature Communications. A revised analysis incorporating σ-bonded materials, advanced stabilization approaches, and anisotropic effects would better align with the journal's standards for transformative research.

My major concerns about this work:
The author may neglect of σ-bonding electron metallization. The study overlooks the potential of σ-electron metallization, a promising pathway highlighted in prior research (e.g., Li2B3C, Li3B4C2, predicted Tc>50K) σ-electrons exhibit strong electron-phonon coupling due to their directional bonding, yet the dataset appears limited to hydrides and boron-doped carbons, omitting σ-dominated systems. This narrow scope undermines the claim of "comprehensive" analysis.
While the authors note the instability of high-Tc candidates (e.g., Li2AgH6 lies 0.319eV/atom above the convex hull), they fail to propose viable stabilization strategies. For instance, pressure quenching or chemical doping—successfully employed in high-pressure hydrides (e.g., LaH10) are not explored. Without actionable synthesis routes, the practical relevance of predictions remains questionable.
The dataset excludes emerging material classes like σ-bonded superconductors and recent experimental breakthroughs (e.g., sulfur hydrides under moderate pressure). Additionally, the isotropic approximation for Tc calculation risks underestimating anisotropic effects critical in multi-gap systems (e.g., MgB2) This raises doubts about the generalizability of conclusions.

Reviewer #2

(Remarks to the Author)
The paper "The Maximum Tc of Conventional Superconductors at Ambient Pressure" presents an in-depth theoretical analysis of the upper limits for the critical temperature (Tc) of conventional superconductors under ambient pressure. Using a dataset of over 20,000 materials, the authors explore the relationship between electron-phonon coupling, phonon frequencies, and superconductivity, ultimately concluding that achieving room-temperature superconductivity under ambient pressure is highly unlikely. While the work is solid in its analysis, several key areas could benefit from further consideration.

Comments:
1) While the dataset of 20,000 materials is substantial, it remains relatively small compared to the vast number of potential structures needed to converge a ternary convex hull, for example. A clearer discussion of this limitation would help set realistic expectations for the scope of the study and the general applicability of the dataset. Also, I would also appreciate a better discussion on what separates this work from the other recent publications of the authors utilizing their database, where they already analyze Tc vs Ehull trends etc.

2) The study relies on several approximations, which may not hold true for all materials and could influence the results. To just mention one example, some of the authors argued that non-Lorentzian phonon line shapes (Comm. Phys. 7:150, 2024), could provide a boost to Tc, particularly in materials with high-frequency phonons. I think the current work would benefit from a more in-depth analysis of the involved approximations and effects beyond that could affect superconductivity.

3) There are additional factors not considered in the current analysis or dataset that could significantly impact the results: For example, low-dimensional materials often exhibit enhanced electron-phonon coupling due to reduced symmetry or dimensionality, and could represent potential candidates for higher-Tc superconductivity. Furthermore, recent advances in pressure quenching experiments, such as those by Deng et al. (PNAS 118(28), 2021, and PNAS 122(6), 2025), show promise for stabilizing high-Tc materials under pressure and quenching them to ambient conditions. (These are again just two examples)

5) The authors do a good job presenting the theoretical framework, but the paper would benefit from a more explicit acknowledgment of the limitations of the dataset and the models used. While the study concludes that room-temperature conventional superconductivity at ambient pressure is extremely unlikely, it is important to recognize that this conclusion is based on current models, the dataset and assumptions. As computational methods improve and new experimental techniques emerge, the potential for unexpected breakthroughs remains, and this possibility should be more clearly addressed.

In my (subjective) opinion, the pursuit of room-temperature superconductivity, while extremely challenging, is still a worthy goal, and a more open-ended perspective on future developments in this area would be warranted.

To summarize, I recommend revision of the article before considering publication.

minor comments:
Why did the authors choose the (modified) McMillan formula instead of the Allen-Dynes McMillan expression with strong-coupling correction factors? For the regions of el-ph coupling the authors are mostly interested in (lambda > 1.5) the Allen-Dynes expression should provide a closer match to the actual Migdal-Eliashberg theory.

I'm surprised the authors did not cite Dolui et al., PRL 132, 166001 (2024) in the discussion on Mg2IrH6 and related compounds. Also, do the authors think that the different mu* is the only reason between the significantly different predicted values for Mg2IrH6 between Sanna et al., npj Comp. Mat. 10, 44 (2024) and Dolui et al., PRL 132, 166001 (2024)?

Equally surprising is the omission of references: Hutcheon et al., PRB 101, 144505 (2020) and Shipley et al. PRB 104, 054501 (2021).

page 3, right column, first paragraph: typo in "... the most important dependence of lambda is om an average ..." what do the authors want to say, I didn't understand this sentence.

Reviewer #3

(Remarks to the Author)
The presented manuscript fits into the current and important research topic concerning the search for superconductors with increasingly higher critical temperatures. The authors assess the possibility of finding a superconductor with a classical, electron-phonon mechanism, whose critical temperature can approach room temperature. Unfortunately, from the point of view of the development of civilization, their predictions are quite pessimistic and the possibilities of finding a material that will superconduct under normal conditions are slim.Their analysis is based on the results of theoretical calculations (within the Eliashberg formalism and the density functional theory for superconductors) performed for a huge number of 20,000 materials. The highest predicted Tc reaches around 100 K in hypothetical materials. They also argue with recently published works, including Ref. 37, which quite optimistically assesses the possibility of existence of systems that under atmospheric pressure will be able to show superconductivity with temperatures close to room temperature. This topic, due to its scientific importance, timeliness and great interest in the scientific community, makes the manuscript, in my opinion, eligible for publication in Nature Communications. The applied state-of-the-art methods and in-depth analysis of the most promising candidates for the highest-Tc materials (SCDFT and anharmonic calculations) leave no doubt as to the reliability of the presented results. However, the manuscript requires some improvements, I list my comments below.
1. Eq. 1, page 2, is actually the Allen-Dynes version of McMillan's formula, as Authors mention later, so proper citations and name should be used from the beginning. The Allen-Dynes PRB 1975 paper is not cited. Also, for large \lambda > 1, the Allen-Dynes formula including correction factors (called f_1, f_2 in the Allen-Dynes paper) should be used, there is no information is this was used in the Tc calculations showed in Supplemental Material.
2. There is no information on the value of \mu* used in all Eliashberg-type calculations. \mu* = 0.10 is mentioned in some cases. Also, for the chosen systems where SCDFT calculations were done the calculated mu* could be given (or the "Allen-Dynes" equivalent which would give the same Tc as the SCDFT calculations but within the Allen-Dynes formula)
3. Technical details of the calculations are given only for Li2AgH6 and Li2AuH6. I suggest giving them also for other systems shown in SI.
4. The calculations and predictions presented in the paper are based on the conventional electron-phonon coupling mechanism, as of course underlined by the Authors. However, the electron-phonon interaction can also go beyond the "classical" Eliashberg picture, for example the chiral phonon-electron interaction. I wonder what is the Authors opinion of the

possibility that this can lead to comparable (100 K) or higher Tc?

Version 1:

Reviewer comments:

Reviewer #2

(Remarks to the Author)
I appreciate the detailed replies to the comments given by the authors and the added information brought to the manuscript. In its revised form, I can support publication of this manuscript in Nature Communications.

I would have two further questions to the authors:
1.) How was configurational entropy considered for materials with 3 or more different constituent elements?
2.) In their reply letter, the authors mention that their machine-learned model has "[...] now an error below 1K [...]". Is this the mean error or the absolute, and what's the reference it is compared against?

Reviewer #3

(Remarks to the Author)
In my opinion the Authors have answered to all remarks and have improved their manuscript. I recommend publication of the manuscript in the current form.

Open Access This Peer Review File is licensed under a Creative Commons Attribution 4.0 International License, which permits use, sharing, adaptation, distribution and reproduction in any medium or format, as long as you give appropriate credit to the original author(s) and the source, provide a link to the Creative Commons license, and indicate if changes were made.
In cases where reviewers are anonymous, credit should be given to 'Anonymous Referee' and the source.
The images or other third party material in this Peer Review File are included in the article’s Creative Commons license, unless indicated otherwise in a credit line to the material. If material is not included in the article’s Creative Commons license and your intended use is not permitted by statutory regulation or exceeds the permitted use, you will need to obtain permission directly from the copyright holder.
To view a copy of this license, visit https://creativecommons.org/licenses/by/4.0/

REVIEWER #1

The study leverages a dataset of over 20,000 metals to statistically analyze the interplay between electron-phonon coupling (λ) and logarithmic average phonon frequencies (ωlog). This provides a rare empirical foundation for estimating the upper limit of Tc in conventional superconductors. The prior assumptions (e.g., McMillan's formula) by demonstrating that the optimal α^2F(ω) function for maximizing T_c is unphysical due to the inherent trade-off between ωlog and λ. This refines the understanding of phonon-mediated superconductivity. While the work provides valuable insights into conventional superconductivity, its exclusion of σ-electron systems, insufficient synthesis strategies, and dataset limitations significantly weaken the novelty and impact. These issues, compounded by the speculative nature of proposed materials, render the manuscript not suitable for publication in Nature Communications. A revised analysis incorporating σ-bonded materials, advanced stabilization approaches, and anisotropic effects would better align with the journal's standards for transformative research.

Reply: Thank you for the valuable report and thorough review of our manuscript. We are grateful for your detailed feedback and are pleased to address your concerns, as we believe our work makes significant contributions that warrant publication.

My major concerns about this work: The author may neglect of σ-bonding electron metallization. The study overlooks the potential of σ-electron metallization, a promising pathway highlighted in prior research (e.g., Li_2B_3C, $Li_3B_4C_2$, predicted $T_c > 50$K) σ-electrons exhibit strong electron-phonon coupling due to their directional bonding, yet the dataset appears limited to hydrides and boron-doped carbons, omitting σ-dominated systems. This narrow scope undermines the claim of "comprehensive" analysis.

Reply: Thank you for raising this important point. We believe there may be a misunderstanding regarding our dataset, and we appreciate the opportunity to clarify this matter.
All σ-electron systems that the referee refers to were already contained in our dataset. We included most of the materials that have been proposed in the literature as conventional high-T_c superconductors, though many required manual addition into the database, since they are often thermodynamically highly unstable and were therefore absent from Alexandria.
The systems that we have considered include a series of materials based on MgB_2 structure [1–12], on the diamond structure [13–18], cage-like structures [19–30], hydrides [31–34], and many other compounds [35–40]. In several of these families, we also performed exhaustive machine-learning accelerated [41] high-throughput calculations that went well beyond what was already reported in the literature. For example, we studied (for a X and Y running over the periodic table) all possible XY_2 compounds in the structure of MgB_2; XBC in the structure of LiBC [11]; $MgXB_4$ compounds in the structure of $MgAlB_4$ [12]; all possible B–C phases in the diamond structure for unit cells with up to 12 atoms, all possible $Li_xB_yC_z$ structures with up to 12 atoms in the unit cell, all XB_3N_3 and XYB_6C_6 filled sodalite compounds [20], etc. In this revised version of the manuscript we have improved on the description of the dataset, including the information above and the associated references on page 2.

While the authors note the instability of high-T_c candidates (e.g., Li_2AgH_6 lies 0.319 eV/atom above the convex hull), they fail to propose viable stabilization strategies. For instance, pressure quenching or chemical doping—successfully employed in high-pressure hydrides (e.g., LaH_{10}) are not explored. Without actionable synthesis routes, the practical relevance of predictions remains questionable.

Reply: We thank the Referee for this important question regarding synthesis strategies. We believe this comment actually highlights the central thesis and primary contribution of our work.
The literature is indeed replete with proposed "stabilization strategies" for high-temperature superconductors that have consistently failed to materialize in experimental practice. This persistent gap between theoretical predictions and experimental reality has had significant deleterious consequences for the field, leading some researchers to question the reliability of computational approaches for conventional superconductivity [42] and materials stability assessment. Also in this case, a statistical analysis provides crucial empirical grounding for understanding why many of these stabilization attempts fail. For a material like Li_2AgH_6 that lies at 0.319 eV/atom above the convex hull, the probability of successful stabilization at ambient conditions is vanishingly small from a statistical perspective, as there are simply no known experimental examples of materials with such extreme thermodynamic instability being successfully synthesized and stabilized through conventional approaches, like pressure quenching or chemical doping. Note that

the "experimental" compounds in materials databases that are classified as very unstable by DFT calculations are usually related to mistakes or misinterpretations of experimental data. Note also, that many of the theoretically proposed high-temperature superconductors (see above) are as far from the hull as Li_2AgH_6 or even further, including some of the σ-compounds that the referee mentions.
However, compounds with large thermodynamic instability retain significant academic and practical value even if they cannot be experimentally realized, as they allow us to understand how very high electron-phonon coupling and superconducting transition temperatures could be realized in a realistic atomic system.
We included a discussion on stabilization by quenching on page 6.

The dataset excludes emerging material classes like σ-bonded superconductors and recent experimental breakthroughs (e.g., sulfur hydrides under moderate pressure).

Reply: Regarding σ-bonded superconductors, as detailed in our previous response, these systems are extensively represented in our dataset across multiple structural families. For sulfur hydrides and other systems under moderate pressure, our analysis is far more comprehensive than the reviewer suggests (this is now properly stated in the manuscript): 1. We did study most of the compounds that have been proposed in the literature. However, the vast majority of these hydride structures turn out to have dynamical instabilities at ambient pressure. 2. We used our machine-learning workflow to examine more than 100 million compounds including 3 million hydrides. We then performed calculations for almost 40,000 of these hydrides with density functional perturbation theory to find that 33,000 of these (corresponding to 85% of the calculated systems) had imaginary frequencies in their phonon spectrum. The remaining 15% are included in the dataset we present in the manuscript.
We hope that the referee understands that describing each of the hundreds of thousands of compounds investigated individually would be impractical within a manuscript format. However, all our datasets are publicly available at https://alexandria.icams.rub.de/ allowing interested readers to search for any specific compounds or families. Upon acceptance of this manuscript, we will naturally update these databases with our complete analysis. In the revised manuscript, we have significantly expanded our description of the dataset to include additional references to specific material families already explored in literature (on page 2).

Additionally, the isotropic approximation for T_c calculation risks underestimating anisotropic effects critical in multi-gap systems (e.g., MgB_2) This raises doubts about the generalizability of conclusions.

Reply: The reviewer raises an important point that we have systematically addressed in our analysis.
The isotropic approximation can be a significant limitation, particularly for multi-gap systems. In view of that, we have been performing anisotropic calculations, either by solving the anisotropic Eliashberg equations or through density-functional theory for superconductors. For all compounds that appear at the top of our T_c list, anisotropic effects turn out to be very minor, and do not affect T_c significantly. This points to the fact that our best compounds are already "fully optimized" (see below for further discussion), and T_c has indeed reached its maximum value. Of course, for compounds with a lower T_c there is a larger margin for optimization. This can be seen from a recent preprint from the Marzari group [43] where they performed anisotropic calculations for 242 materials and did find a significant increase in the T_c of some compounds. The values that they find, however, are still well below our suggested maximum T_c of around 100 K, in agreement with our arguments.
We have clarified also this point in the revised manuscript (point 2 on page 5).

Pressure quenching or chemical doping—successfully employed in high-pressure hydrides (e.g., LaH_{10}) are not explored.

Reply: We appreciate the opportunity to address both pressure quenching and chemical doping approaches in detail. We acknowledge that pressure quenching is an established technique that has shown some success in superconductivity research. For example, it has recently been successfully used to quench a superconducting phase of the topological alloy $Bi_{0.5}Sb_{1.5}Te_3$ [44] (quenched from 4 GPa, achieving T_c =10 K at ambient pressure), FeSe (quenched from 4.15 GPa, reaching T_c =37 K at ambient pressure [45]), and Sb (quenched from 10.9 GPa, retaining a T_c of 3 K at ambient pressure). The clathrate SrB_3C_3, synthesized under high pressure, shows superconductivity with a maximum T_c of 22 K at 23 GPa [24]. Even if the authors of the latter work argue that their sample survives to ambient pressure, it was not possible to measure superconductivity at ambient pressure.
However, a critical pattern emerges from successful examples: they involve relatively modest pressures and contain heavy atoms. The fundamental challenge for hydride systems lies in the extreme mobility of hydrogen atoms and the

requirement for stabilization starting from hundreds of GPa. The probability of successfully quenching such systems is, in our assessment, vanishingly small due to the inherent kinetic instability of hydrogen in these structures.
We have included this discussion in the manuscript on page 6.
To conclude, we would like to note that our materials database does not include only materials *on* the convex hull but also contains a multitude of unstable and metastable compounds at ambient conditions. Also these compounds were screened for superconductivity and are included in the present manuscript. It is clear that ALEXANDRIA is biased towards thermodynamically stable compounds, but it is also clear that these low-energy materials are those with realistic synthesis prospects, even using out-of-equilibrium methods such as pressure quenching.
In what concerns chemical doping, this is a quite different and complex issue. The referee is right in stating that we did not systematically explore chemical doping. Let us divide this discussion in two parts: a) standard doping, where a small percentage of dopant atoms is added, removed or substituted, in an attempt to move the Fermi level to a region of higher density of states or softening some phonons to a sweet spot in frequency. In a first approximation this can be achieved by a rigid shift of the Fermi energy in the calculations. We have tried this for the compounds in the top of our list with partial success (see, e.g., Mg_2PdH_6 and Mg_2PtH_6 [46]). We observed that materials with high critical temperature are usually already in a near-optimal configuration, and in this case the T_c can not be significantly improved. This is the case of, e.g., MgB_2 [47] or Mg_2IrH_6 [46]. Of course, other compounds with lower values of T_c have a larger potential for optimization, but they will not yield record T_c. b) degenerate doping, where a significant amount of atoms is added, removed or substituted. Indeed this often leads to substantial increase of T_c, as can be seen, for example, in Ref. [25]. However, this kind of systems are to a large extent already represented in our dataset, and should therefore be easily predicted by our machine-learning model.
Therefore we firmly believe that while doping is a crucial technique to improve superconducting properties, a more systematical exploration of doping would not significantly affect our statistical analysis of the maximum T_c. Such a discussion is now included in the manuscript (point 3 on page 5).

REVIEWER #2

The paper "The Maximum T_c of Conventional Superconductors at Ambient Pressure" presents an in-depth theoretical analysis of the upper limits for the critical temperature (T_c) of conventional superconductors under ambient pressure. Using a dataset of over 20,000 materials, the authors explore the relationship between electron-phonon coupling, phonon frequencies, and superconductivity, ultimately concluding that achieving room-temperature superconductivity under ambient pressure is highly unlikely. While the work is solid in its analysis, several key areas could benefit from further consideration.

Reply: We thank the Reviewers for the thoughtful overview of our manuscript and for recognizing the solid foundation of our analysis.

Comments: 1) While the dataset of 20,000 materials is substantial, it remains relatively small compared to the vast number of potential structures needed to converge a ternary convex hull, for example. A clearer discussion of this limitation would help set realistic expectations for the scope of the study and the general applicability of the dataset.

Reply: We believe there may be some misunderstanding about the true scale of our analysis, and we welcome the opportunity to provide clarification.
We would like to stress that 20,000 is the number of dynamically stable materials for which we have calculated electron-phonon coupling and superconducting properties. Using the machine learning model, which has now an error below 1 K, we have by now screened more than 100 million compounds, including almost 3 million hydrides (this is now clearly stated in the revised manuscript for clarity). For all compounds predicted by the machine learning model to have a T_c above 10 K, we have performed full electron-phonon calculations. We also performed successive cycles of training the model and predicting T_c, creating an iterative refinement process that has exhaustively explored the chemical space.
Although the machine learning model is not perfect, our extensive calculations strongly suggest that a superconductor with a T_c much larger than 100 K is extremely unlikely to exist in chemical systems accessible at ambient pressure. First, the error of our machine learning model has converged to below 1 K, indicating that we have captured the essential physics of electron-phonon coupling in these materials. Second, all our recent batches consistently fail to find any new superconductors with very large T_c (> 50 K), despite our material database growing at 1–2 million

compounds per week. This plateau in discoveries, despite exponentially increasing data, provides strong statistical evidence that we have identified the fundamental upper limits of superconducting transition temperatures.

Furthermore, our dataset comprehensively covers the chemical space relevant to superconductivity: it includes binary, ternary, quaternary, and higher multinary compounds with careful sampling of stoichiometries, covers the entire periodic table with particular focus on light elements that maximize phonon frequencies, and explicitly explores hydrides which have shown the highest T_c values under pressure. The convergence of our results across multiple independent approaches (different machine learning architectures, iterative refinement, and exhaustive chemical space exploration) gives us high confidence in our conclusions.

However, we acknowledge the referee's point and have added sentences explaining the inherent limitations of any finite dataset, particularly regarding the challenges of fully covering ternary and higher-order convex hulls (point 1 on page 5)

Also, I would also appreciate a better discussion on what separates this work from the other recent publications of the authors utilizing their database, where they already analyze T_c vs Ehull trends etc.

Reply: While it is true that some of us have previously analyzed distributions of superconducting properties (λ, $\omega_{\log}$, T_c) for smaller datasets of around 8000 compounds [48], studied specific families of superconducting compounds [49–51], and examined individual high-T_c hydrides [52, 53], the current manuscript represents a significant conceptual and methodological advancement.

Furthermore, and equally importantly, we have increased considerably the size of our dataset, particularly for high-T_c compounds that are relevant for the discussion of the maximum T_c.

Specifically:

1. We added to the dataset all relevant compounds that were proposed in the literature as high-T_c superconductors. We also performed exhaustive calculations for some of the most promising families of high-T_c compounds (please see the reply to Referee 1 for more information).

2. We performed more cycles of our workflow (batch of electron-phonon calculations, retraining of the machine learning model, prediction of T_c with the machine). Although our machine still finds new superconductors with medium values of T_c, no new superconductor has appeared with record (or near record) T_c for a long time.

We included this description on page 5.

This manuscript thus represents not merely an incremental expansion, but a qualitative leap toward providing definitive, statistically-grounded answers to one of the most important questions in superconductivity research. The confidence we now have in our maximum T_c estimates, derived from this comprehensive approach, enables us to make authoritative statements about the realistic prospects for room-temperature conventional superconductivity that were not possible in our earlier, more limited studies.

We also note that recent claims of possible conventional superconductity at 300–600 K in the literature further motivated us to write the present manuscript.

2) The study relies on several approximations, which may not hold true for all materials and could influence the results. To just mention one example, some of the authors argued that non-Lorentzian phonon line shapes (Comm. Phys. 7:150, 2024), could provide a boost to T_c, particularly in materials with high-frequency phonons. I think the current work would benefit from a more in-depth analysis of the involved approximations and effects beyond that could affect superconductivity.

Reply: The reviewer raises valid concerns that we have systematically addressed in our analysis. We acknowledge that our study involves several approximations that could potentially influence the results. In the revised manuscript, we now include a comprehensive discussion of possible effects and approximations that might impact our conclusions, including anisotropy (see reply to Referee 1), doping (see reply to Referee 1), anharmonicity (see below), reduced dimensionality effects (see the answer to next question), treatment of Coulomb interaction (see below), and dataset completeness with machine learning model errors (see above). These additions can be found on page 5 and 6.

In particular, the referee is pointing to the possible impact of using anharmonic spectral functions in the calculations instead of delta functions centered at the harmonic or anharmonic phonon frequencies, as discussed in the Comm. Phys. article. The calculation of the critical temperature performed with the anharmonic spectral function is bounded from below by the T_c obtained with auxiliary SSCHA phonons and from above by the T_c obtained with SSCHA Hessian phonons. We have checked for the Li_2AuH_6 case, by calculating T_c with the isotropic Migdal-Eliashberg equations and μ^*=0.1, that the T_c calculated by the full spectral function would be bounded between 128 and 144 K. Thus, it does not seem that the impact of anharmonic spectral functions on T_c will be large in this system.

In addition of this specific example, we may consider more in general the effect on anharmonicity for the maximum possible T_c. It is true that anharmonic effects are crucial for many systems. However, their main effect is in the stabilization of otherwise unstable or nearly unstable phases. This is, for example, what is observed in H_3S with the stabilization of the cubic phase at lower pressures than with the harmonic approximation [54]. Furthermore, the stabilization of soft-modes usually leads to a decrease of λ and consequent decrease of T_c as, for example, in H_3S under high pressure [54] or in HPd at ambient pressure [55].
Concerning the treatment of Coulomb interaction, this quantity is often handled at the level of μ^* approximation, and this is also the case for the data in figs. 2 and 4. The standard value of $\mu^* = 0.10$ (that we also adopt) is likely a lower limit for real systems, and it is often a gross underestimation for compounds with very high values of T_c. For example, for Mg_2IrH_6 [46] we computed the Coulomb interaction from first principles, and found that the resulting repulsion is extremely large at the Fermi level (yielding a $\mu = 0.58$), consistently with the large electronic density of states. Furthermore, as the density of states decreases away from the Fermi level, the Coulomb renormalization mechanism becomes very inefficient. A direct calculation of μ^* is quite difficult, but a rough estimation indicates that the value should be $\mu^* \gg 0.25$, much larger than the 0.10 value commonly used, hence leading to a considerable decrease of T_c.

> 3) There are additional factors not considered in the current analysis or dataset that could significantly impact the results: For example, low-dimensional materials often exhibit enhanced electron-phonon coupling due to reduced symmetry or dimensionality, and could represent potential candidates for higher-T_c superconductivity. Furthermore, recent advances in pressure quenching experiments, such as those by Deng et al. (PNAS 118(28), 2021, and PNAS 122(6), 2025), show promise for stabilizing high-T_c materials under pressure and quenching them to ambient conditions. (These are again just two examples)

Reply: With regard to quenching please see the reply to referee 1 (and additional text in the manuscript on page 6). Concerning reduced symmetry, it is true that our initial high-throughput studies concentrated on high-symmetry space groups [48]. However, since then we removed all constrains from the search, and we now include all compounds, including some with space group P1. Curiously, and in agreement with Matthias, many (most) of the higher-T_c compounds are very symmetric, and often cubic.
Concerning reduced dimensionality, recently some of us applied our approach to 2D systems [56], motivated by the hypothesis that reduced dimensionality could enhance transition temperatures. However, the values of T_c for 2D systems turned out to be systematically lower than for 3D compounds. In retrospect, this outcome can be understood through fundamental physical principles. First, 2D systems exhibit systematically lower electronic densities of states at the Fermi level compared to 3D materials. Second, the frequencies of out-of-plane phonon modes are characteristically reduced in layered structures. While this frequency reduction does lead to an increase in the electron-phonon coupling parameter λ, it simultaneously causes a substantial decrease in the logarithmically averaged phonon frequency $\omega_{\log}$. According to the McMillan formula, the net effect of these competing factors results in an overall reduction of T_c, despite the enhanced coupling strength. We have included this discussion in the manuscript (page 6, point 5).

> 5) The authors do a good job presenting the theoretical framework, but the paper would benefit from a more explicit acknowledgment of the limitations of the dataset and the models used. While the study concludes that room-temperature conventional superconductivity at ambient pressure is extremely unlikely, it is important to recognize that this conclusion is based on current models, the dataset and assumptions. As computational methods improve and new experimental techniques emerge, the potential for unexpected breakthroughs remains, and this possibility should be more clearly addressed.

Reply: We have included a long discussion on the shortcomings of our approach, and how they might influence the maximum T_c. The concept expressed by the Referee is certainly true, in the sense that there will always be space for a breakthrough, e.g. the discovery of a new mechanism for superconductivity. However our claim is that high values of T_c are not to be expected within a *conventional mechanism*, which is precisely the type of mechanism that we include in our simulations. Our level of accuracy do not leave much space for significant improvement, therefore we uphold our claim that it will be very unlikely to obtain a stable conventional superconductor with T_c significantly higher than 100 K.

> In my (subjective) opinion, the pursuit of room-temperature superconductivity, while extremely challenging, is still a worthy goal, and a more open-ended perspective on future developments in this area would be warranted.

Reply: We want to emphasize that we share the excitement and hope for discovering room-temperature superconductivity - it would represent one of the most transformative scientific breakthroughs of our time, and we would be absolutely delighted to be proven wrong in our assessment.
Our conclusions are not driven by pessimism but by our responsibility to provide the scientific community with the most rigorous, data-driven analysis possible. The statistical evidence from our comprehensive dataset of over 100 million screened compounds consistently points toward fundamental physical limits around 100 K for conventional superconductors at ambient pressure.
However, we do not exclude that room-temperature superconductivity could be achieved through unconventional pairing mechanisms, high pressure conditions, or entirely new physical phenomena that transcend current theoretical frameworks. Our hope is that this analysis helps guide future research toward the most promising avenues while establishing realistic expectations about the fundamental limits of conventional electron-phonon mediated superconductivity.
We included an open-ended perspective in the conclusions.

To summarize, I recommend revision of the article before considering publication.

minor comments: Why did the authors choose the (modified) McMillan formula instead of the Allen-Dynes McMillan expression with strong-coupling correction factors? For the regions of el-ph coupling the authors are mostly interested in (lambda > 1.5) the Allen-Dynes expression should provide a closer match to the actual Migdal-Eliashberg theory.

Reply: In the workflow to calculate T_c we actually used Allen-Dynes's modified McMillan formula, the original McMillan formula (Eq.1, page 2) is only used for sake of simplicity in the discussion and for the contours plot in Fig. 2. Note that the Allen-Dynes formula depends not only on $\omega_{\log}$ and λ but also on ω_2, so it is not possible to use it to generate contours in Fig. 2. This has been clarified in the manuscript.

I'm surprised the authors did not cite Dolui et al., PRL 132, 166001 (2024) in the discussion on Mg_2IrH_6 and related compounds. Also, do the authors think that the different μ^* is the only reason between the significantly different predicted values for Mg_2IrH_6 between Sanna et al., npj Comp. Mat. 10, 44 (2024) and Dolui et al., PRL 132, 166001 (2024)? Equally surprising is the omission of references: Hutcheon et al., PRB 101, 144505 (2020) and Shipley et al. PRB 104, 054501 (2021).

Reply: We have added the references suggested by the referee, together with a few more references regarding machine-learning exploration of superconductors. Concerning the disagreement between the transition temperatures, we have identified two concurring reasons for such a large disagreement. First in Ref. [57] the estimated electron-phonon coupling is 16% larger than ours ($\lambda = 2.5$ against $\lambda = 2.13$). As discussed in the text, the presence of a van Hove singularity at the Fermi level makes the estimation of λ quite sensitive to small electronic and computational details (in particular the smearing used), therefore such a difference is not completely unexpected. Second, and most importantly, in Ref. [57] Coulomb interactions are included in the μ^* approximation (assuming $\mu^* = 0.16$). We compute the Coulomb interaction from first principles, and we find that the resulting repulsion is extremely large at the Fermi level ($\mu = 0.58$), consistently with the large electronic DOS. Furthermore, as the DOS drops away from the Fermi level, the Coulomb renormalization mechanism is very inefficient. In such a situation a calculation of μ^* is quite difficult, but a rough estimation indicates that the value should be $\mu^* \gg 0.25$, much larger than the value used in Ref. [57].

page 3, right column, first paragraph: typo in "... the most important dependence of lambda is om an average ..." what do the authors want to say, I didn't understand this sentence.

Reply: We changed the sentence to "where it was recognized that λ is inversely proportional to the square of the average phonon frequency". This is Eq. (43) of the famous McMillan paper.

REVIEWER #3

The presented manuscript fits into the current and important research topic concerning the search for superconductors with increasingly higher critical temperatures. The authors assess the possibility of finding a superconductor with a classical, electron-phonon mechanism, whose critical temperature can approach room temperature. Unfortunately, from the point of view of the development of civilization, their predictions are quite pessimistic and the possibilities of finding a material that will superconduct under normal conditions are slim. Their analysis is based on the results of theoretical calculations (within the Eliashberg formalism and the density functional theory for superconductors) performed for a huge number of 20,000 materials. The highest predicted T_c reaches around 100 K in hypothetical materials. They also argue with recently published works, including Ref. 37, which quite optimistically assesses the possibility of existence of systems that under atmospheric pressure will be able to show superconductivity with temperatures close to room temperature. This topic, due to its scientific importance, timeliness and great interest in the scientific community, makes the manuscript, in my opinion, eligible for publication in Nature Communications. The applied state-of-the-art methods and in-depth analysis of the most promising candidates for the highest-T_c materials (SCDFT and anharmonic calculations) leave no doubt as to the reliability of the presented results. However, the manuscript requires some improvements, I list my comments below.

Reply: We thank the Reviewer for their thoughtful and constructive assessment of our manuscript.

1. Eq. 1, page 2, is actually the Allen-Dynes version of McMillan's formula, as Authors mention later, so proper citations and name should be used from the beginning. The Allen-Dynes PRB 1975 paper is not cited. Also, for large $\lambda > 1$, the Allen-Dynes formula including correction factors (called f_1, f_2 in the Allen-Dynes paper) should be used, there is no information is this was used in the T_c calculations showed in Supplemental Material.

Reply: Yes, Eq.1 is the Allen-Dynes version of McMillan formula [58]. We now correctly cite the reference. We indeed used Allen-Dynes formula with correction factors in our workflow to obtain T_c, as mention above in reply to refree #2. We add a clarification to this point in the manuscript.

2. There is no information on the value of μ^* used in all Eliashberg-type calculations. $\mu^* = 0.10$ is mentioned in some cases. Also, for the chosen systems where SCDFT calculations were done the calculated μ^* could be given (or the "Allen-Dynes" equivalent which would give the same T_c as the SCDFT calculations but within the Allen-Dynes formula)

Reply: In this work, the high-throughput estimations of T_c are done with the Allen-Dynes formula using $\mu^* = 0.1$ this is now mentioned more precisely in the manuscript (e.g. in the caption of Fig. 2 and Fig. 4). On the other hand, Eliashberg simulations are done with a new approach which includes the Coulomb interaction without using the μ^* approximation, but using the full energy width of the screened Coulomb matrix elements [59]. Similarly to what is typically done in superconducting-DFT. As mentioned on page 4. this new Eliashberg approach for the case of Li_2AgH_6 gives a T_c very close to the Allen-Dynes approach with $\mu^* = 0.1$, indicating the astonishing accuracy of this old formula. More precisely the fitted value of μ^* to obtain the same T_c is 0.12. However this is not always the case, and in our experience large deviations are expected when the electronic density of states has anomalies (e.g., a band gap close to the Fermi level). Concerning SCDFT, the theory usually gives critical temperatures quite similar to those obtained with the full-Elaishberg approach in the medium to low coupling regime. At extremely high coupling it tends to predict lower values of T_c so in the case of Li_2AgH_6 it would indicate a $\mu^* \simeq 0.18$.

3. Technical details of the calculations are given only for Li_2AgH_6 and Li_2AuH_6. I suggest giving them also for other systems shown in SI.

Reply: The technical details did not change from our previous publication [48, 60]. We now refer the reader to these two references in the caption of Fig. 1.

4. The calculations and predictions presented in the paper are based on the conventional electron-phonon coupling mechanism, as of course underlined by the Authors. However, the electron-phonon interaction can also go beyond the "classical" Eliashberg picture, for example the chiral phonon-electron interaction. I wonder what is the Authors opinion of the possibility that this can lead to comparable (100 K) or higher T_c?

Reply: There are many possibilities to go beyond the conventional electron-phonon interaction. For example one could go beyond the GW diagram in Eliashberg theory and include non-adiabatic phonon contributions (vertex

corrections), spin-orbit effects in the phonon dynamics and electron phonon coupling (leading to chiral phonons) or phonon-magnon interactions. Clearly these are all very interesting research directions and we can only give an opinion about their potential.

Vertex corrections have been studied extensively, and there is not much evidence that violations of Migdal's theorem can truly open up to significant increase of T_c. Spin orbit effect also do not seem that promising. Certainly spin-orbit can affect phonon frequencies and electronic states so it could strongly influence the prediction of a specific material. But in a global sense it seems to lead to a tuning effect and not to a significant new paradigm for the electron-phonon pairing. Therefore, from a statistical point of view, it does not seem to be a game changer on the topic of the maximum T_c. Probably the stronger candidate could be phonon-magnon interaction, especially in the presence of strong anharmonicitiy. In fact the spin paring has clearly the potential to provide large coupling matrix elements, the effectiveness of which could be enhanced by the presence of phonons with large oscillation amplitudes. However this is presently beyond the capabilities of *ab initio* methodologies, and technically would be considered an unconventional mechanism, beyond the topic of the current manuscript.

[1] H. Rosner, A. Kitaigorodsky, and W. E. Pickett, Prediction of high T_c superconductivity in hole-doped LiBC, Phys. Rev. Lett. **88**, 127001 (2002).

[2] P. P. Singh, Hole-doped, high-temperature superconductors Li_xBC, Na_xBC and C_x: a coherent-potential-based prediction, Solid State Commun. **124**, 25 (2002).

[3] M. Gao, Z.-Y. Lu, and T. Xiang, Prediction of phonon-mediated high-temperature superconductivity in $Li_3B_4C_2$, Phys. Rev. B **91**, 045132 (2015).

[4] T. Bazhirov, Y. Sakai, S. Saito, and M. L. Cohen, Electron-phonon coupling and superconductivity in Li-intercalated layered borocarbide compounds, Phys. Rev. B **89**, 045136 (2014).

[5] T.-T. Pham and D.-L. Nguyen, First-principles prediction of superconductivity in MgB_3C_3, Phys. Rev. B **107** (2023).

[6] S. K. Kwon, S. J. Youn, K. S. Kim, and B. I. Min, New high temperature diboride superconductors: AgB_2 and AuB_2, arXiv 10.48550/arXiv.cond-mat/0106483 (2001).

[7] M. J. Mehl, D. A. Papaconstantopoulos, and D. J. Singh, Effects of C, Cu, and Be substitutions in superconducting MgB_2, Phys. Rev. B **64**, 140509 (2001).

[8] A. Islam, F. Parvin, F. Islam, M. Islam, A. Islam, and I. Tanaka, AgB_2: Superconductivity and the role of paramagnons, Physica C **466**, 76 (2007).

[9] H. J. Choi, S. G. Louie, and M. L. Cohen, Prediction of superconducting properties of CaB_2 using anisotropic Eliashberg theory, Phys. Rev. B **80**, 064503 (2009).

[10] M. Calandra, A. N. Kolmogorov, and S. Curtarolo, Search for high T_c in layered structures: The case of LiB, Phys. Rev. B **75**, 144506 (2007).

[11] E. Haque, M. A. Hossain, and C. Stampfl, First-principles prediction of phonon-mediated superconductivity in XBC (X = Mg, Ca, Sr, Ba), Phys. Chem. Chem. Phys. **21**, 8767 (2019).

[12] Y. Li, H. Chen, G. Han, and X. Zhao, First-principles investigation of the superconducting properties of $MgXB_4$ (X = Al, Li, Na, K), Physica C **577**, 1353732 (2020).

[13] S. Saha, S. D. Cataldo, M. Amsler, W. von der Linden, and L. Boeri, High-temperature conventional superconductivity in the boron-carbon system: Material trends, Phys. Rev. B **102** (2020).

[14] M. Calandra and F. Mauri, High-T_c Superconductivity in Superhard Diamondlike BC_5, Phys. Rev. Lett. **101**, 016401 (2008).

[15] Q. Li, H. Wang, Y. Tian, Y. Xia, T. Cui, J. He, Y. Ma, and G. Zou, Superhard and superconducting structures of BC_5, J. Appl. Phys. **108**, 023507 (2010).

[16] P.-J. Chen and H.-T. Jeng, Ambient-pressure high-temperature superconductivity in stoichiometric hydrogen-free covalent compound $BSiC_2$, New J. Phys. **22**, 033046 (2020).

[17] F. J. Ribeiro and M. L. Cohen, Possible superconductivity in hole-doped BC_3, Phys. Rev. B **69**, 212507 (2004).

[18] Z.-F. Ouyang, X.-W. Yan, and M. Gao, Electronic structure, phonons, and high-temperature phonon-mediated superconductivity in lithium-intercalated diamond-like boron compounds, Appl. Phys. Express **13**, 083003 (2020).

[19] D. Hapiuk, M. A. L. Marques, P. Mélinon, S. Botti, B. Masenelli, and J. A. Flores-Livas, Superconductivity in an expanded phase of ZnO: an ab-initio study, New J. Phys. **17**, 043034 (2015).

[20] X. Li, X. Yong, M. Wu, S. Lu, H. Liu, S. Meng, J. S. Tse, and Y. Li, Hard BN clathrate superconductors, J. Phys. Chem. Lett. **10**, 2554 (2019).

[21] J.-N. Wang, X.-W. Yan, and M. Gao, High-temperature superconductivity in SrB_3C_3 and BaB_3C_3 predicted from first-principles anisotropic Migdal-Eliashberg theory, Phys. Rev. B **103** (2021).

[22] S. Di Cataldo, S. Qulaghasi, G. B. Bachelet, and L. Boeri, High-T_c superconductivity in doped boron-carbon clathrates, Phys. Rev. B **105**, 064516 (2022).

[23] T.-T. Gai, P.-J. Guo, H.-C. Yang, Y. Gao, M. Gao, and Z.-Y. Lu, Van Hove singularity induced phonon-mediated superconductivity above 77 K in hole-doped SrB_3C_3, Phys. Rev. B **105**, 224514 (2022).

[24] L. Zhu, H. Liu, M. Somayazulu, Y. Meng, P. A. Guńka, T. B. Shiell, C. Kenney-Benson, S. Chariton, V. B. Prakapenka,

H. Yoon, J. A. Horn, J. Paglione, R. Hoffmann, R. E. Cohen, and T. A. Strobel, Superconductivity in SrB_3C_3 clathrate, Phys. Rev. Res. **5**, 013012 (2023).
[25] N. Geng, K. P. Hilleke, L. Zhu, X. Wang, T. A. Strobel, and E. Zurek, Conventional High-Temperature Superconductivity in Metallic, Covalently Bonded, Binary-Guest C–B Clathrates, J. Am. Chem. Soc. **145**, 1696 (2023).
[26] X. Cui, K. P. Hilleke, X. Wang, M. Lu, M. Zhang, E. Zurek, W. Li, D. Zhang, Y. Yan, and T. Bi, RbB_3Si_3: An Alkali Metal Borosilicide that is Metastable and Superconducting at 1 atm, J. Phys. Chem. C **124**, 14826 (2020).
[27] R. Miao, Z. Bai, C. Liu, F. Wu, X. Zhang, H. Wang, and J. Yang, Potentially high-temperature superconductivity in $K_{1-x}B_6$: A first-principles prediction, Physica C **551**, 16 (2018).
[28] Z. Yuan, L. Hao, K. Luo, M. Xiong, C. Shao, F. Ling, and D. Yu, Structural stability, electronic structure, and superconductivity of cubic sodium hexaboride NaB_6 from first-principle calculations, Chem. Phys. Lett. **722**, 80 (2019).
[29] S. Chen, H. Xie, D. Xu, J. Chen, B. Cao, M. Liang, Y. Sun, X. Gai, X. Wang, M. Yang, M. Zhang, D. Duan, D. Li, and F. Tian, Superconductivity of cubic MB_6 (M = Na, K, Rb, Cs), J. Chem. Phys. **160**, 044702 (2024).
[30] P. Zhang, Y. Tian, Y. Yang, H. Liu, and G. Liu, Stable Rb-B compounds under high pressure, Phys. Rev. Res. **5**, 013130 (2023).
[31] S. Yu, X. Jia, G. Frapper, D. Li, A. R. Oganov, Q. Zeng, and L. Zhang, Pressure-driven formation and stabilization of superconductive chromium hydrides, Sci. Rep. **5** (2015).
[32] H. Tran and T. N. Vu, Machine-learning approach for discovery of conventional superconductors, Phys. Rev. Mater. **7**, 054805 (2023).
[33] Y. He, J. Lu, X. Wang, and J. jie Shi, Phonon-mediated superconductivity in the metal-bonded perovskite Al_4H up to 54 K under ambient pressure, Phys. Rev. B **108** (2023).
[34] Y. He and J.-j. Shi, Few-Hydrogen High-T_c Superconductivity in $(Be_4)_2H$ Nanosuperlattice with Promising Ductility under Ambient Pressure, Nano Lett. **23**, 8126 (2023).
[35] W. Pickett, B. Klein, and D. Papaconstantopoulos, Theoretical prediction of MoN as a high T_c superconductor, Physica B+C **107**, 667 (1981).
[36] A. D. Hernández, J. A. Montoya, G. Profeta, and S. Scandolo, First-principles investigation of the electron-phonon interaction in OsN_2: Theoretical prediction of superconductivity mediated by N-N covalent bonds, Phys. Rev. B **77** (2008).
[37] D.-H. Wang, H.-Y. Zhou, C.-H. Hu, A. R. Oganov, Y. Zhong, and G.-H. Rao, BaC: a thermodynamically stable layered superconductor, Phys. Chem. Chem. Phys. **16**, 20780 (2014).
[38] D.-L. Nguyen, C.-R. Hsing, and C.-M. Wei, Theoretical prediction of superconductivity in monolayer CoO_2, Nanoscale **11**, 17052 (2019).
[39] L. Hao, X. Li, Y. Zhang, K. Luo, Y. Gao, F. Ling, Y. Wu, Z. Zhao, and D. Yu, Prediction of Li_2B novel phases and superconductivity under varying pressures, Comput. Mater. Sci. **158**, 255 (2019).
[40] K. Choudhary and K. Garrity, Designing high-T_c superconductors with BCS-inspired screening, density functional theory, and deep-learning, npj Comput. Mater. **8**, 244 (2022).
[41] J. Schmidt, N. Hoffmann, H.-C. Wang, P. Borlido, P. J. M. A. Carriço, T. F. T. Cerqueira, S. Botti, and M. A. L. Marques, Machine-Learning-Assisted Determination of the Global Zero-Temperature Phase Diagram of Materials, Adv. Mater. **35**, 2210788.
[42] J. E. Hirsch, Superconducting materials: Judge and jury of bcs-electron–phonon theory, Appl. Phys. Lett. **121**, 080501 (2022), https://pubs.aip.org/aip/apl/article-pdf/doi/10.1063/5.0104968/20036996/080501_1_5.0104968.pdf.
[43] M. Bercx, S. Poncé, Y. Zhang, G. Trezza, A. G. Ghezeljehmeidan, L. Bastonero, J. Qiao, F. O. von Rohr, G. Pizzi, E. Chiavazzo, and N. Marzari, Charting the landscape of bardeen-cooper-schrieffer superconductors in experimentally known compounds, arXiv 10.48550/arXiv.2503.10943 (2025).
[44] L. Deng, B. Wang, C. Halbert, D. J. Schulze, M. Gooch, T. Bontke, T.-W. Kuo, X. Shi, S. Song, N. Salke, H.-D. Yang, Z. Ren, R. J. Hemley, E. Zurek, R. P. Prasankumar, and C.-W. Chu, Creation, stabilization, and investigation at ambient pressure of pressure-induced superconductivity in $Bi_{0.5}Sb_{1.5}Te_3$, Proc. Natl. Acad. Sci. U.S.A. **122**, e2423102122 (2025).
[45] L. Deng, T. Bontke, R. Dahal, Y. Xie, B. Gao, X. Li, K. Yin, M. Gooch, D. Rolston, T. Chen, Z. Wu, Y. Ma, P. Dai, and C.-W. Chu, Pressure-induced high-temperature superconductivity retained without pressure in FeSe single crystals, Proc. Natl. Acad. Sci. U.S.A. **118**, e2108938118 (2021).
[46] A. Sanna, T. F. T. Cerqueira, Y.-W. Fang, I. Errea, A. Ludwig, and M. A. L. Marques, Prediction of ambient pressure conventional superconductivity above 80 K in hydride compounds, npj Comput. Mater. **10**, 44 (2024).
[47] G. Simon and M. Miryala, Impact of doping on mgb2 superconductors: A comprehensive review, J. Alloys Compd. Commun. **3**, 100023 (2024).
[48] T. F. Cerqueira, A. Sanna, and M. A. Marques, Sampling the materials space for conventional superconducting compounds, Adv. Mater. **36**, 2307085 (2024).
[49] N. Hoffmann, T. F. T. Cerqueira, P. Borlido, A. Sanna, J. Schmidt, and M. A. L. Marques, Searching for ductile superconducting Heusler X_2YZ compounds, npj Comput. Mater. **9**, 138 (2023).
[50] N. Hoffmann, T. F. Cerqueira, J. Schmidt, and M. A. Marques, Superconductivity in antiperovskites, npj Comput. Mater. **8**, 150 (2022).
[51] Đ. Dangić, Y.-W. Fang, T. F. T. Cerqueira, A. Sanna, M. A. L. Marques, and I. Errea, Ambient pressure high temperature superconductivity in $RbPH_3$ facilitated by ionic anharmonicity, arXiv 10.48550/arXiv.2411.03822 (2024).
[52] A. Sanna, T. F. T. Cerqueira, Y.-W. Fang, I. Errea, A. Ludwig, and M. A. L. Marques, Prediction of ambient pressure conventional superconductivity above 80 K in hydride compounds, npj Comput. Mater. **10**, 44 (2024).
[53] T. F. Cerqueira, Y.-W. Fang, I. Errea, A. Sanna, and M. A. Marques, Searching materials space for hydride superconductors at ambient pressure, Adv. Funct. Mater. **34**, 2404043 (2024).

[54] I. Errea, M. Calandra, C. J. Pickard, J. Nelson, R. J. Needs, Y. Li, H. Liu, Y. Zhang, Y. Ma, and F. Mauri, High-pressure hydrogen sulfide from first principles: A strongly anharmonic phonon-mediated superconductor, Phys. Rev. Lett. **114**, 157004 (2015).

[55] I. Errea, M. Calandra, and F. Mauri, First-principles theory of anharmonicity and the inverse isotope effect in superconducting palladium-hydride compounds, Phys. Rev. Lett. **111**, 177002 (2013).

[56] T. H. B. da Silva, T. Cavignac, T. F. T. Cerqueira, H.-C. Wang, and M. A. L. Marques, Machine-learning accelerated prediction of two-dimensional conventional superconductors, Mater. Horiz. **12**, 3408 (2025).

[57] K. Dolui, L. J. Conway, C. Heil, T. A. Strobel, R. P. Prasankumar, and C. J. Pickard, Feasible route to high-temperature ambient-pressure hydride superconductivity, Phys. Rev. Lett. **132**, 166001 (2024).

[58] P. B. Allen and R. C. Dynes, Transition temperature of strong-coupled superconductors reanalyzed, Phys. Rev. B **12**, 905 (1975).

[59] C. Pellegrini, R. Heid, and A. Sanna, Eliashberg theory with ab-initio coulomb interactions: a minimal numerical scheme applied to layered superconductors, J. Phys.: Mater. **5**, 024007 (2022).

[60] T. F. T. Cerqueira, Y.-W. Fang, I. Errea, A. Sanna, and M. A. L. Marques, Searching materials space for hydride superconductors at ambient pressure, Adv. Funct. Mater. **34**, 2404043 (2024).

RESPONSE TO REVIEWERS' COMMENTS

We thank both reviewers for their positive feedback and support for publication. We are pleased to address the additional questions raised by Reviewer #2.

REVIEWER #2

> I appreciate the detailed replies to the comments given by the authors and the added information brought to the manuscript. In its revised form, I can support publication of this manuscript in Nature Communications.

We thank the reviewer for their positive assessment and recommendation for publication.

> I would have two further questions to the authors: 1.) How was configurational entropy considered for materials with 3 or more different constituent elements?

We appreciate this important question. In our study, we focus exclusively on ordered crystalline compounds, as these are the materials contained in the Alexandria database. While the database does include crystals that differ in site configuration, we do not perform thermodynamic treatments to obtain ensemble averages of electronic and superconducting properties. We believe the reviewer may be referring to the calculation of superconducting properties in disordered systems. To our knowledge, this challenging problem has been addressed in only a limited number of studies, notably the work by Di Cataldo et al. on Ti-Nb alloys [1] and the recent contribution by Christopher Heil [2]. Based on current understanding, we anticipate that thermodynamic averaging of and $\alpha^2F(\omega)$ would be unlikely to lead to significant increases in T_c values. However, we acknowledge that this represents an interesting avenue for future investigation and appreciate the reviewer for bringing attention to this important consideration. We have added a comment and these references to our revised manuscript.

> 2.) In their reply letter, the authors mention that their machine-learned model has "[...] now an error below 1K [...]". Is this the mean error or the absolute, and what's the reference it is compared against?

The ≈1 K error corresponds to the mean absolute error (MAE) in the unbiased testing set of Ref. [3].

REVIEWER #3

> In my opinion the Authors have answered to all remarks and have improved their manuscript. I recommend publication of the manuscript in the current form.

Reply: We thank the reviewer for their positive assessment and recommendation for publication.

---

[1] A. Cucciari, D. Naddeo, S. Di Cataldo, and L. Boeri, Nbti: A nontrivial puzzle for the conventional theory of superconductivity, Phys. Rev. B **110**, L140502 (2024).

[2] P. Ferreira, R. Lucrezi, I. Guilhon, M. Marques, L. Teles, C. Heil, and L. Eleno, Ab initio modeling of superconducting alloys, Mater. Today Phys. **48**, 101547 (2024).

[3] J. Schmidt, N. Hoffmann, H.-C. Wang, P. Borlido, P. J. M. A. Carriço, T. F. T. Cerqueira, S. Botti, and M. A. L. Marques, Machine-Learning-Assisted Determination of the Global Zero-Temperature Phase Diagram of Materials, Adv. Mater. **35**, 2210788.
